# Cascade C–H Halogenation of Tetraazapyrene with Iodine Halides

**DOI:** 10.1021/acs.orglett.5c02966

**Published:** 2025-10-07

**Authors:** Mingming Li, Xinyi Liu, Silvio Decurtins, Shi-Xia Liu

**Affiliations:** Department of Chemistry, Biochemistry and Pharmaceutical Sciences, W. Inäbnit Laboratory for Molecular Quantum Materials and WSS-Research Center for Molecular Quantum Systems, 27210 University of Bern, Freiestrasse 3, 3012 Bern, Switzerland

## Abstract

Iodine halide as an efficient and selective halogenation reagent is presented for the cascade halogenation of an electron-deficient tetraazapyrene (TAP), yielding both chlorinated and heterohalogenated derivatives. This approach enables precise control over the LUMO energy levels of the resulting TAPs by varying the type and extent of halogenation, offering a powerful strategy for fine-tuning their electronic properties. These findings open new avenues for the rational design of TAP-based materials in organic molecular electronics.

## Graphical abstract

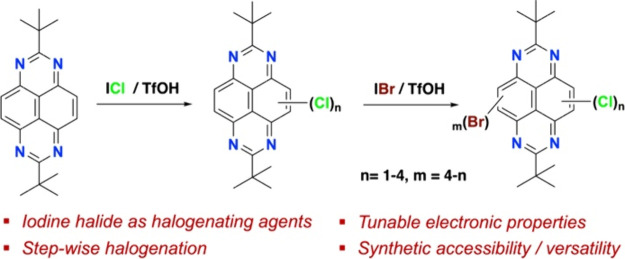

Polycyclic aromatic hydrocarbons (PAHs) such as anthracene and pyrene act as nanoscale fragments of graphene and play a vital role in the bottom-up synthesis of graphene nanostructures with atomic precision due to their structural planarity, thermal stability, and easy halogenation.^1^ The electronic properties of the resulting graphene-like nanomaterials are strongly affected by many factors, including size, shape, edge configuration, and heteroatom insertion, which can be adjusted by chemical design of the corresponding PAH precursors.^2^ As a consequence, the rational synthesis of PAH building blocks is of high importance for advancing molecular optoelectronics, spintronics, and quantum information technologies.^3^ Among these, N-embedded PAHs are particulary appealling as a result of their intrinsic optoelectronic and electrochemical properties induced by the incorporation of nitrogen atoms into the π-conjugated framework leading to a low-lying LUMO which facilitates enhanced electron affinity and charge transport characteristics.^2c–2e,4^

As a prototypical N-PAH, 1,3,6,8-tetraazapyrene (TAP) stands out due to its unique structural symmetry and pronounced electron-deficient character induced by its high nitrogen content. These features not only enhance its electron affinity but also promote n-type semiconducting behavior, making TAP a promising candidate for high-performance organic electronic devices.^5,6^ Our recent studies have shown that the self-assembly of 4,5,9,10-tetrabromo-TAP leads to the formation of stable radical species on both Ag(111)^7^ and superconducting Pb(111) surfaces.^8^ Remarkably, switchable topological superconductors in combination with a 2D spin–lattice can be realized,^8b^ which is of paramount importance for the construction of topological quantum bits. However, the influence of the molecular electronic structure and the substituents on the TAP core on the charging pattern on surfaces remains elusive. On the other hand, selective halogenation of the TAP scaffold appears to be challenging, which in turn limits its application in advanced optoelectronic materials.

To bridge the gap between molecular chemistry and quantum physics and further explore TAP-based organic functional materials, we aim to develop an efficient cascade C–H halogenation of the TAP scaffold for the synthesis of chlorinated and heterohalogenated TAP derivatives. Our approach allows us to systematically investigate the impact of the number and type of halogen atoms on the TAP core on the LUMO energy levels.

The previously reported synthesis of 4,5,9,10-tetrachloro-TAP derivatives either requires harsh conditions such as dissolving TAP in chlorosulfonic acid, followed by a reaction with highly toxic chlorine gas in the presence of a catalytic amount of iodine, or can only be accomplished on a small scale with a low yield of 13% even when a large amount of dichloroisocyanuric acid in concentrated sulfuric acid is used at 100 °C over a prolonged reaction time of 10 days.^5c^ Moreover, sequential chlorination on the TAP core remains elusive.

In response to these issues, we have developed an efficient and facile synthetic protocol for the cascade chlorination of the TAP core, using only iodine monochloride (ICl) in trifluoromethanesulfonic acid (TfOH). As illustrated in Scheme 1, a reaction of 2,7-di-*tert*-butyl-1,3,6,8-tetraazapyrene (^***t***^ **Bu-TAP**)^9^ with 1 equiv of ICl in TfOH at 80 °C overnight afforded a mixture of mono-, di-, and trichlorinated products. The analytically pure **Cl-** ^***t***^ **Bu-TAP** (37%), **4,10-Cl**_**2**_ **-** ^***t***^ **Bu-TAP** (18%), and **Cl**_**3**_ **-** ^***t***^ **Bu-TAP** (5%) were then obtained by careful column chromatography. It is worth noting that both the 4,9- and 4,10-disubstituted isomers were formed during the halogenation process, likely in a ratio of approximately 2:98, as confirmed by the atomic occupancy of the Cl1 atom over two positions in the X-ray structure of **4,10-Cl**_**2**_ **-** ^***t***^ **Bu-TAP** (Figure S1 and Table S9). Importantly, they were successfully isolated as analytically pure compounds by column chromatography, as evidenced by distinct chemical shifts in their ^1^H NMR spectra. The 4,9-isomer, however, was obtained only in trace amounts (approximately 1% yield, Table S1) while the 4,5-isomer was not detected, indicating that further substitution at the 5-position is likely suppressed due to the reduced reactivity at this site once the 4-position is already substituted.

**Scheme 1:**
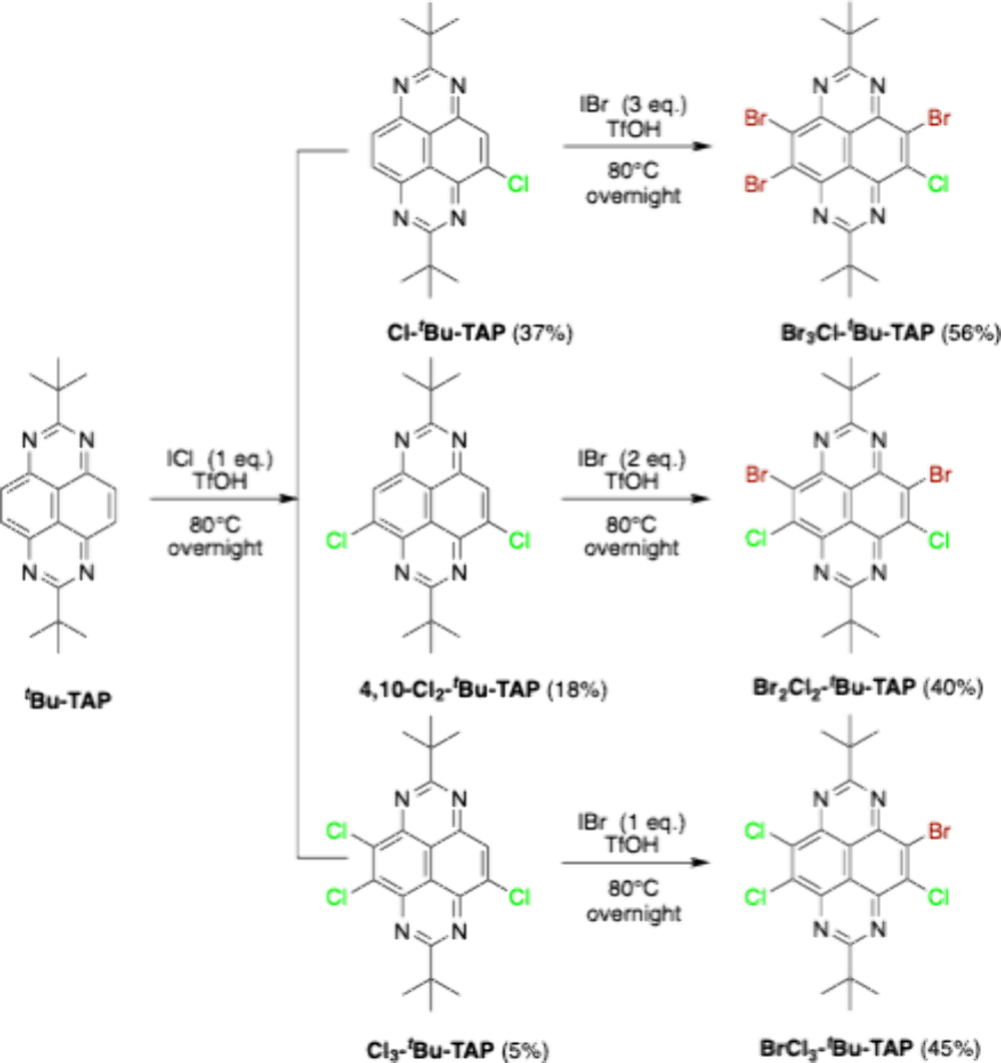
Synthetic Routes to a Series of Chlorinated and Hetero-Halogenated TAP Derivatives

Notably, increasing the amount of ICl led to a significant shift in the distribution of chlorinated products, with a higher proportion of the 3- and 4-fold chlorinated TAPs, and a corresponding decrease in the mono- and disubstituted products (Table S1). It turns out that the optimal yields of 22%, 38%, and 80% for **4,10-Cl**_**2**_ **-** ^***t***^ **Bu-TAP**, **Cl**_**3**_ **-** ^***t***^ **Bu-TAP**, and **Cl**_**4**_ **-** ^***t***^ **Bu-TAP** are achieved by using 2, 3, and 10 equiv of ICl, respectively. Upon the addition of 3 equiv of ICl, the combined yield of different chlorinated products reaches a maximum of approximately 80%. However, when the reaction was perfomed with ICl (3 equiv) in acetic acid, only monochlorinated compound **Cl-** ^***t***^ **Bu-TAP** was produced as a yellow powder in a yield of 42%. The results obtained differ significantly from what we initially expected, given that ICl is typically used as an iodination reagent for PAHs via electrophilic substitution.^10^ There are only a few reports on its use as a chlorination reagent, which is however limited to electron-rich PAHs with up to three fused phenyl rings, such as anthracene and phenanthrene.^11^ The proposed reaction mechanism usually involves an initial electron transfer, leading to the formation of a transient complex PAH^·+^ICl^·–^, which then either collapses via an ion-pair interaction for chlorination or a radical-pair interaction for iodination.^11d^ Since TAP is electron-deficient and difficult to oxidize to its radical cation, the reaction proceeds through an alternative mechanism. Bearing in mind that chlorination efficiency is dramatically enhanced in strong acid TfOH compared to the weak acetic acid, it is anticipated that the carbocation at the TAP core is initiated and stabilized through protonation of the N atoms. Subsequent nucleophilic attack by chloride (Cl^–^), followed by iodine cation-mediated oxidative rearomatization, yields the chlorinated TAPs.

The chlorination reaction using 10 equiv of ICl was further optimized by varying the reaction time and temperature. It was found that complete 4-fold chlorination of ^***t***^ **Bu-TAP** was achieved within 4 h, affording **Cl**_**4**_ **-** ^***t***^ **Bu-TAP** in a 78% yield, which is comparable to the yield of 80% obtained by overnight reaction under the same conditions.

As expected, at room temperature the reaction proceeded more slowly. After 3 h, only **Cl**_**3**_ **-** ^***t***^ **Bu-TAP** was formed. When the reaction mixture was subsequently heated up to 60 °C and stirred for another 2 h, a mixture consisting predominantly of **Cl**_**3**_ **-** ^***t***^ **Bu-TAP** and a small amount of **Cl**_**4**_ **-** ^***t***^ **Bu-TAP** was observed. Raising the temperature further to 100 °C did not accelerate the reaction either; complete conversion to **Cl**_**4**_ **-** ^***t***^ **Bu-TAP** still required 3 h. These results indicate that longer reaction times were required for complete chlorination at temperatures below 80 °C, pointing out the high reaction efficiency specifically at 80 °C.

To fine-tune the LUMO energy levels of halogenated TAPs, a similar strategy was applied to further brominate partially chlorinated TAPs. Instead of using ICl, 3 equiv of IBr was reacted overnight with **Cl-** ^***t***^ **Bu-TAP** in TfOH at 80 °C to afford **Br**_**3**_ **Cl-** ^***t***^ **Bu-TAP** in a 56% yield (Scheme 1). Both **Br**_**2**_ **Cl**_**2**_ **-** ^***t***^ **Bu-TAP** and **BrCl**_**3**_ **-** ^***t***^ **Bu-TAP** were successfully synthesized by reacting the corresponding chlorinated precursor with 2 and 1 equiv of IBr, respectively. There is no doubt that iodine halide proved to be an efficient halogenation reagent for TAP, facilitating a cascade halogenation which allows us to prepare a series of heterohalogenated TAP derivatives. These newly prepared compounds were unambiguously characterized by ^1^H and ^13^C NMR spectroscopy and high-resolution mass spectrometry. All analytical data were consistent with their proposed chemical structures. Particularly, single crystals of **Cl**_**2**_ **-** ^***t***^ **Bu-TAP** were obtained by slowly evaporating its solution in CH_2_Cl_2_, and the structure of **4,10-Cl**_**2**_ **-** ^***t***^ **Bu-TAP** was elucidated by X-ray diffraction analysis of a single crystal (Figure S1, Table S2).

To study the impact of both the number and type of halogen substituents on the LUMO energy levels of ^***t***^ **Bu-TAP** derivatives, their electrochemical properties were investigated using cyclic voltammetry and differential pulse voltammetry in CH_2_Cl_2_ (Table 1, Figures S2–S10). In the negative potential window, ^***t***^ **Bu-TAP**, **Cl-** ^***t***^ **Bu-TAP**, and **Cl**_**2**_ **-** ^***t***^ **Bu-TAP** undergo one reversible reduction process at −1.21 V, −1.09 V, and −1.01 V, respectively. In contrast, both **Cl**_**3**_ **-** ^***t***^ **Bu-TAP** and **Cl**_**4**_ **-** ^***t***^ **Bu-TAP** show two reversible reductions (Figures S2–S6), corresponding to the successive reduction of TAP to its radical anion and dianion species. The first reduction potential shifts positively as the number of chlorine atoms on the TAP core increases, indicating a gradual decrease in the LUMO energy level (approximately 0.07 eV per Cl atom, Table 1). Notably, **Cl**_**4**_ **-** ^***t***^ **Bu-TAP** has a LUMO level that is reduced by 0.33 eV compared to ^***t***^ **Bu-TAP**. This observation can be attributed to the strong electron-withdrawing inductive effect of chlorine atoms. However, the consecutive replacement of Cl with Br has a negligible influence on the LUMO energy level, with even a slight increase of 0.01 eV per Br. This subtle change is very probably due to the pronounced p−π conjugation between the lone pairs on the Br atoms and the TAP π-system. Furthemore, **Br**_**4**_ **-** ^***t***^ **Bu-TAP**,^9,12^ **Br**_**3**_ **Cl-** ^***t***^ **Bu-TAP**, and **Br**_**2**_ **Cl**_**2**_ **-** ^***t***^ **Bu-TAP** display a single reversible reduction wave at −0.90 V, −0.80 V, and −0.86 V, respectively, while **BrCl**_**3**_ **-** ^***t***^ **Bu-TAP** undergoes two reversible reductions (Figures S7–S10). These results indicate that the incorporation of Br atoms can significantly inhibit the further reduction of the radical anions to dianion species, which in turn can be attributed to the electron-donating effect of Br.

**Table 1:** Summary of Electrochemical Data, HOMO and LUMO Energy Levels and Optical Bandgap (*E*_g_) of a Series of Chlorinated and Hetero-Halogenated TAP Derivatives and Redox Potentials (V) vs Ag/AgCl in CH_2_Cl_2_

compound	*E*_1/2_^red1^ (V)	*E*_1/2_^red2^ (V)	*E*_onset_^red1^ (eV)	LUMO (eV)^a^	HOMO (eV)^b^	*E*_g_ (eV)^c^
^***t***^ **Bu-TAP**	–1.21		–1.00	–3.34	–6.56	3.22
**Cl-** ^***t***^ **Bu-TAP**	–1.09		–0.93	–3.41	–6.58	3.17
**Cl**_**2**_ **-** ^***t***^ **Bu-TAP**	–1.01		–0.86	–3.48	–6.61	3.13
**Cl**_**3**_ **-** ^***t***^ **Bu-TAP**	–0.86	–1.53	–0.73	–3.61	–6.74	3.13
**Cl**_**4**_ **-** ^***t***^ **Bu-TAP**	–0.77	–1.36	–0.67	–3.67	–6.78	3.11
**BrCl**_**3**_ **-** ^***t***^ **Bu-TAP**	–0.79	–1.44	–0.67	–3.67	–6.75	3.08
**Br**_**2**_ **Cl**_**2**_ **-** ^***t***^ **Bu-TAP**	–0.86		–0.68	–3.66	–6.76	3.10
**Br**_**3**_ **Cl-** ^***t***^ **Bu-TAP**	–0.80		–0.69	–3.65	–6.75	3.10
**Br**_**4**_ **-** ^***t***^ **Bu-TAP**	–0.90		–0.71	–3.63	–6.69	3.06

a LUMO energy level is calculated from the onset of the first reduction potential according to the equation *E*_LUMO_ = −*e*(*E*_red_^onset^ – 0.46 + 4.8) eV. 4.8 eV is the energy level of ferrocene below the vacuum level. The oxidation potential of Fc/Fc^+^ against Ag/AgCl was recorded to be 0.46 V.

b *E*_HOMO_ = *E*_LUMO_ – *E*_g_.

c Determined from the onset of the lowest energy electronic absorptions in the corresponding UV–vis spectra in solution.

No redox activity was observed for any ^***t***^ **Bu-TAP** derivatives in the positive potential window, indicating that these compounds function exclusively as π-electron acceptors.

The electronic absorption spectra of ^***t***^ **Bu-TAP** derivatives, recorded in CH_2_Cl_2_, are depicted in Figure 1. The most important feature is a series of strong (ε ≈ 1–2 × 10^4^ M^–1^ cm^–1^) and broad absorption bands in the range between 280 and 410 nm, which extends into the visible part of the optical spectrum, as evidenced by their yellow color. The further intense absorption band is observed in the UV region of the spectrum at energies above 280 nm.

**Figure 1:**
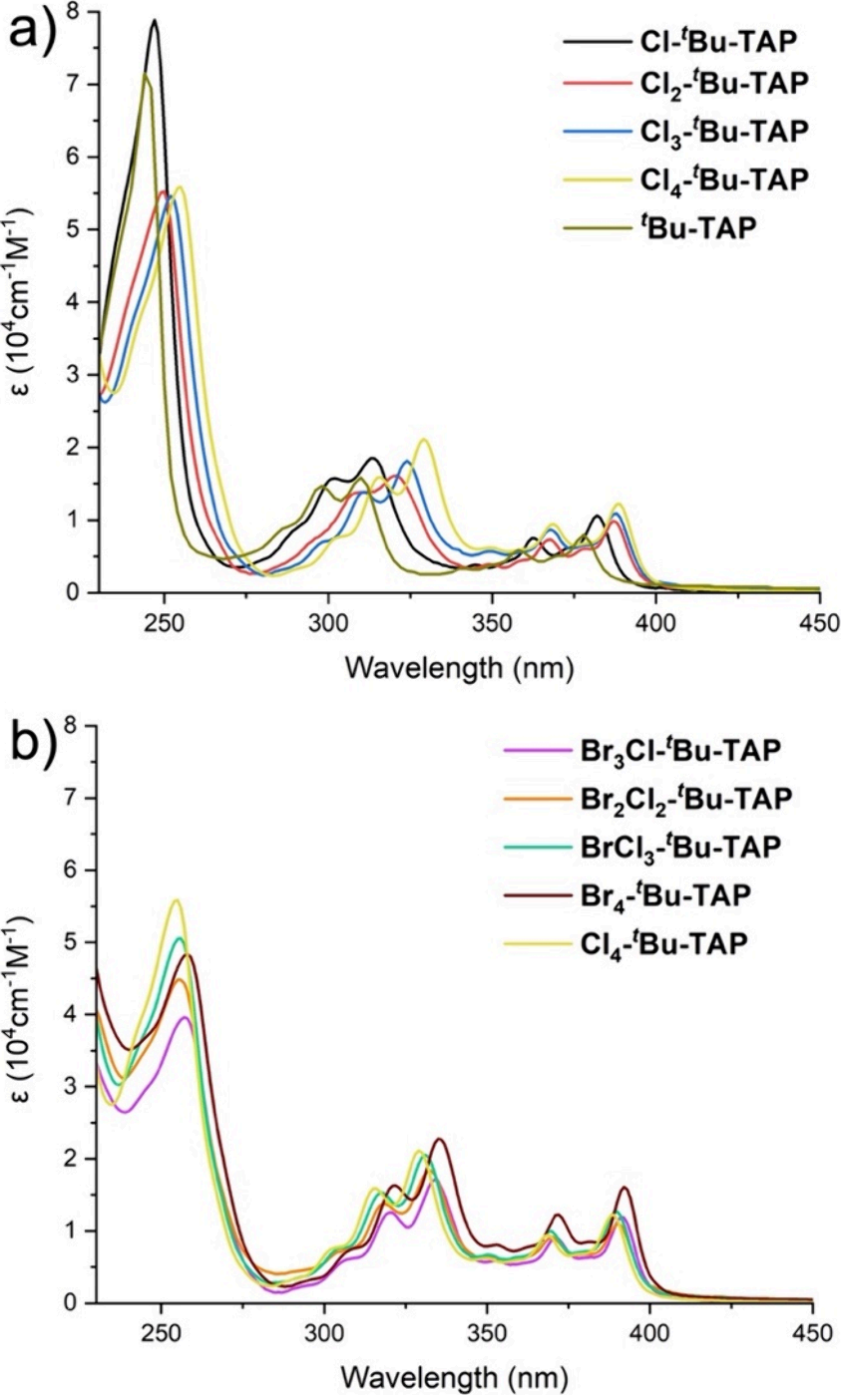
UV–vis absorption spectra of a series of chlorinated (a) and heterohalogenated (b) ^***t***^ **Bu-TAP** derivatives in CH_2_Cl_2_ at rt.

As shown in Figure 1a, the incorporation of Cl atoms leads to substantial bathochromic shifts of π–π* transitions within the 280–410 nm range. As the number of chlorine atoms increases, the lowest-energy absorption band is gradually red-shifted, resulting in a slight reduction of the optical HOMO–LUMO band gap (*E*_g_) (Table 1). A direct comparison of **Cl**_**4**_ **-** ^**t**^ **Bu-TAP** (yellow) to **Br**_**4**_ **-** ^**t**^ **Bu-TAP** (brown) clearly demonstrates that replacing Cl with Br gives rise to noticeable bathochromic shifts of the absorption bands (Figure 1b) and therefore to a decrease in *E*_g_ from 3.11 to 3.06 eV (Table 1). Similar spectral changes were reported in the 4-fold brominated and chlorinated PAHs.^13^ These results are primarily assigned to the opposing effects of Br and Cl. The extent of p−π conjugation and the delocalization of π-electrons across the TAP and halogen atoms is expected to decrease in the order from iodine to fluorine as I is the largest and least electronegative halogen, typically showing the strongest effect. Conversely, the electron-withdrawing inductive effect of halogens increases in the order from I to F, with F being the most electronegative halogen, thereby showing the strongest effect. As a result, the HOMO and LUMO energy levels and *E*_g_ values of three heterohalogenated ^**t**^ **Bu-TAP** derivatives remain nearly unchanged (Table 1). This observation is consistent with the results of the aforementioned CV measurements.

In conclusion, we have successfully developed a highly efficient and straightforward protocol for the cascade halogenation of electron-deficient TAP, enabling the synthesis of a wide variety of chlorinated and heterohalogenated TAP derivatives using iodine halide (bromide and chloride) in TfOH. Through comprehensive UV–vis absorption spectroscopy and electrochemical studies, we demonstrated that the LUMO energy levels of these ^**t**^ **Bu-TAP** derivatives can be precisely tuned by varying the type and extent of halogenation. This tunability, combined with their ability to engage in strong intermolecular interactions, particularly π–π stacking, hydrogen bonding, and halogen bonding, makes them attractive candidates for applications in organic field-effect transistors, organic photovoltaics, as well as active components in molecular spintronics and quantum information processing. Moreover, the halogenated TAP scaffolds can readily undergo C–C couplings such as Heck, Suzuki, and Sonogashira reactions,^5c,14^ allowing facile construction of extended π-conjugated TAP frameworks with tailored functionalities. This work not only offers a robust strategy for the selective functionalization of TAPs but also paves the way for their application in advanced molecular architectures. Our findings underscore the importance of molecular engineering for optimizing electronic properties tailored to specific device requirements. Ultimately, our approach lays the foundation for the development of high-performance materials in organic electronics, with promising implications for future quantum information technologies.

## Supplementary Material



## Data Availability

The data underlying this study are openly available in the published article and its Supporting Information.

## Notes

Experimental details and characterization of all compounds including CV and DPV spectra as well as NMR and MS spectra (PDF)

## †

Hebei Key Laboratory of Organic Functional Molecules, College of Chemistry and Materials Science, Hebei Normal University, Shijiazhuang 050024, China
