# Supplementary material for: Cascade C–H Halogenation of Tetraazapyrene with Iodine Halides
Source: Org Lett. 2025 Oct 7;27(41):11434–9. doi: 10.1021/acs.orglett.5c02966 (PMC12538590; doi:10.1021/acs.orglett.5c02966)
Supplement: Supplementary file 1 [file ol5c02966_si_001.pdf]

Supplementary Information

Cascade C−H Halogenation of Tetraazapyrene with Iodine Halides

*Mingming Li,† Xinyi Liu, Silvio Decurtins, and Shi-Xia Liu**

Department of Chemistry, Biochemistry and Pharmaceutical Sciences, W. Inäbnit Laboratory for Molecular Quantum Materials and WSS-Research Center for Molecular Quantum Systems, University of Bern, Freiestrasse 3, 3012 Bern, Switzerland.

*Correspondence to: shi-xia.liu@unibe.ch

Table of Contents

1. Experimental Section……..........………………………………………………...............S1

2. X-Ray Diffraction Analysis of 4,10-Cl_2-^{t}Bu-TAP………………………………...............S5

3. CV and DPV Spectra……………………………...S15

4. NMR and MS Spectra..…...........................S19

5. References...S35

Experimental Section

General

Chemicals used for the synthesis of the compounds were purchased from commercial suppliers (Sigma-Aldrich, TCI or Alfa Aesar). 2,7-di-tert-butyl-1,3,6,8-tetraazapyrene (**^{t}Bu-TAP**) and **Br_4-^{t}Bu-TAP** were prepared as described in the literature.[1]

UV-Vis absorption spectra were recorded on a Varian Cary-100 Bio-UV/VIS. ^{1}H and ^{13}C NMR spectra were recorded on a Bruker Avance 400 spectrometer at 400 MHz and 101 MHz, respectively. Chemical shifts are reported in parts per million (ppm) and are referenced to the residual solvent peak ($CDCl_3$, δ ^{1}H = 7.26 ppm, δ ^{13}C = 77.16 ppm). High resolution mass spectra (HR MS) were obtained on a Thermo Fisher LTQ Orbitrap XL using Nano Electrospray Ionization.

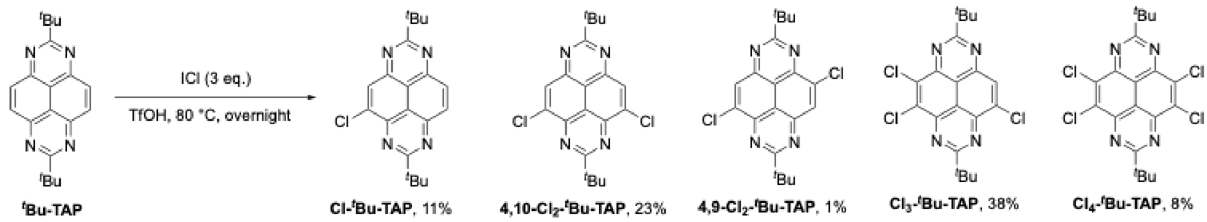

Chlorination of ^{t}Bu-TAP. **^{t}Bu-TAP** (159 mg, 0.5 mmol) was dissolved in CF_3SO_3H (10 mL). Iodine monochloride (244 mg, 1.5 mmol) was added. The reaction mixture was stirred at 80 °C in an oil bath overnight and then allowed to cool to room temperature. The mixture was poured on ice, neutralized with a saturated aqueous solution of Na_2CO_3, and extracted with CH_2Cl_2 (3 × 30 mL). The organic layer was dried over sodium sulfate and filtered. The solvent was removed under reduced pressure, and the residue was purified by column chromatography (silica, hexane/CH_2Cl_2 8:1v/v for **Cl_4-^{t}Bu-TAP**, hexane/CH_2Cl_2 6:1 v/v for **Cl_3-^{t}Bu-TAP**, hexane/CH_2Cl_2 4:1 v/v for **Cl_2-^{t}Bu-TAP**, hexane/CH_2Cl_2 2:1 v/v for **Cl-^{t}Bu-TAP**) to afford the target product as a light-yellow powder.

Cl-^{t}Bu-TAP (19 mg, 11%, a light-yellow powder). ^{1}H NMR (400 MHz, $CDCl_3$) δ 8.68 (s, 1H), 8.55 (s, 2H), 1.70 (s, 9H), 1.66 (s, 9H). ^{13}C NMR (101 MHz, $CDCl_3$) δ 177.35, 177.00, 153.07, 152.57, 152.17, 149.60, 141.37, 136.32, 135.94, 134.59, 112.49, 110.10, 41.00, 40.74, 30.40. HR-MS (ESI, positive): *m/z* $[M + H]^+$ Calcd for $C_{20}H_{22}ClN_4$ 353.1528; Found: 353.1522.

4,10-Cl_2-^{t}Bu-TAP (44 mg, 23%, a light-yellow powder). ^{1}H NMR (400 MHz, $CDCl_3$) δ 8.68 (s, 2H), 1.72 (s, 9H), 1.64 (s, 9H). ^{13}C NMR (101 MHz, $CDCl_3$) δ 177.96, 177.29, 152.01, 150.13, 141.37, 134.89, 112.92, 110.13, 41.34, 40.83, 30.36, 30.33. HR-MS (ESI, positive): *m/z* $[M + H]^+$ Calcd for $C_{20}H_{21}Cl_2N_4$ 387.1138; Found: 387.1132.

4,9-Cl_2-^{t}Bu-TAP (2 mg, 1%, a light-yellow powder). ^{1}H NMR (400 MHz, $CDCl_3$) δ 8.96 (s, 2H), 1.68 (s, 18H). ^{13}C NMR (101 MHz, $CDCl_3$) δ 177.78, 153.07, 150.29, 138.28, 133.36, 111.66, 41.08, 30.34. HR-MS (ESI, positive): *m/z* $[M + H]^+$ Calcd for $C_{20}H_{21}Cl_2N_4$ 387.1138; Found: 387.1135.

Cl_3-^{t}Bu-TAP (80 mg, 38%, a light-yellow powder). ^{1}H NMR (400 MHz, $CDCl_3$) δ 8.70 (s, 1H), 1.71 (s, 9H), 1.67 (s, 9H). ^{13}C NMR (101 MHz, $CDCl_3$) δ 178.15, 177.85, 152.43, 150.21, 150.03, 149.59, 141.67, 139.83, 139.39, 134.67, 111.44, 110.01, 41.41, 41.15, 30.32, 30.30. HR-MS (ESI, positive): *m/z* $[M + H]^+$ Calcd for $C_{20}H_{20}Cl_3N_4$ 421.0748; Found: 421.0742.

Cl_4-^{t}Bu-TAP (18 mg, 8%, a light-yellow powder). ^{1}H NMR (400 MHz, $CDCl_3$) δ 1.71 (s, 18H). ^{13}C NMR (101 MHz, $CDCl_3$) δ 178.30, 150.03, 139.70, 109.92, 41.48, 30.27. HR-MS (ESI, positive): *m/z* $[M + H]^+$ Calcd for $C_{20}H_{19}Cl_4N_4$ 455.0358; Found: 455.0365.

Table S1. Chlorination reaction of ^{t}Bu-TAP with different amount of ICl either in TfOH or acetic acid.

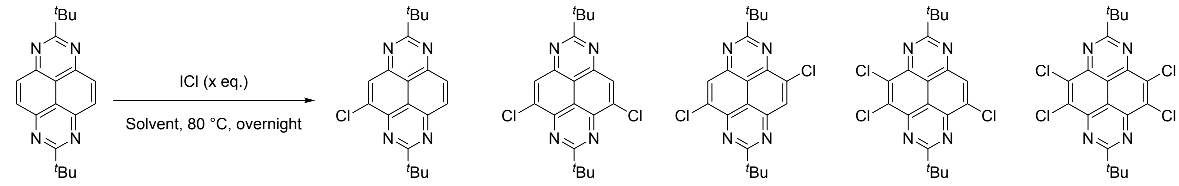

^{t}Bu-TAP **159 mg, 0.5 mmol** **Cl-^{t}Bu-TAP** **4,10-Cl_2-^{t}Bu-TAP** **4,9-Cl_2-^{t}Bu-TAP** **Cl_3-^{t}Bu-TAP** **Cl_4-^{t}Bu-TAP**

Entry	Solvent	ICl (eq.)	**Cl-^{t}Bu-TAP**	**4,10-Cl_2-^{t}Bu-TAP**	**4,9-Cl_2-^{t}Bu-TAP**	**Cl_3-^{t}Bu-TAP**	**Cl_4-^{t}Bu-TAP**	combined yield (%)
1	TfOH	1	65 mg, 37%	35 mg, 18%	1.9 mg, 1%	11 mg, 5%	--	61
2	TfOH	2	55 mg, 31%	42 mg, 22%	2.3 mg, 1%	46 mg, 22%	--	76
3	TfOH	3	19 mg, 11%	44 mg, 23%	2.4 mg, 1%	80 mg, 38%	18 mg, 8%	81
4	TfOH	6				46 mg, 22 %	143 mg, 61%	83
5	TfOH	10	--		--	--	188 mg, 80%	80
6	AcOH	3	70 mg, 42%		--	--	--	42

Optimization of the chlorination reaction of ^{t}Bu-TAP by adjusting the reaction time

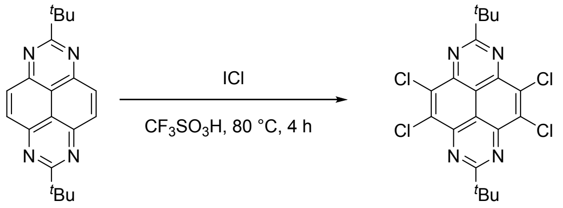

^{t}Bu-TAP **Cl_4-^{t}Bu-TAP**, 78%

^{t}Bu-TAP (50 mg, 0.157 mmol) was dissolved in CF_3SO_3H (10 mL). Iodine monochloride (255 mg, 1.57 mmol) was added. The reaction mixture was heated in an oil bath to 80 °C and stirred for 1 h. Thin layer chromatography (TLC) analysis at this stage revealed a mixture consisting predominantly of **Cl_3-^{t}Bu-TAP**, along with a small amount of **Cl_4-^{t}Bu-TAP**. After 4 h, TLC monitoring showed complete consumption of **^{t}Bu-TAP** and full conversion to **Cl_4-^{t}Bu-TAP**, indicating completion of the reaction. The reaction mixture was allowed to cool down to room temperature, poured on ice, neutralized with a saturated aqueous solution of Na_2CO_3, and extracted with CH_2Cl_2 (3 × 30 mL). The organic layer was dried over sodium sulfate and filtered. The solvent was removed under reduced pressure, and the residue was purified by column chromatography (silica, hexane/CH_2Cl_2 8:1, v/v) to afford **Cl_4-^{t}Bu-TAP** as a light-yellow powder (56 mg, 78%).

Optimization of the chlorination reaction of ^{t}Bu-TAP by adjusting the reaction temperature

^{t}Bu-TAP (50 mg, 0.157 mmol) was dissolved in CF_3SO_3H (10 mL). Iodine monochloride (255 mg, 1.57 mmol) was added. The reaction mixture was stirred at room temperature for 3 h, TLC analysis indicated the exclusive formation of **Cl_3-^{t}Bu-TAP**. The temperature was then increased to 60 °C in an oil bath, after an additional 2 h, only a tiny amount of **Cl_4-^{t}Bu-TAP** was observed. Subsequently, the reaction was heated up to 100 °C in an oil bath and kept for another 3 h, TLC analysis at this stage showed complete conversion to **Cl_4-^{t}Bu-TAP**, with no remaining **^{t}Bu-TAP** detected. The mixture was then cooled down to room temperature, poured on ice, neutralized with a saturated aqueous solution of Na_2CO_3, and extracted with CH_2Cl_2 (3 × 30 mL). The organic layer was dried over sodium sulfate and filtered. The solvent was removed under reduced pressure, and the residue was purified by column chromatography (silica, hexane/CH_2Cl_2 8:1, v/v) to afford **Cl_4-^{t}Bu-TAP** as a light-yellow powder (58 mg, 80%).

Scale-up of the chlorination reaction of ^{t}Bu-TAP

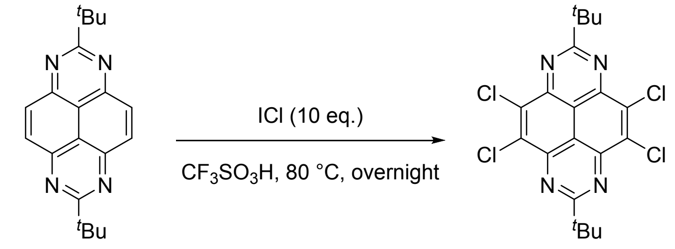

1 mmol, 318 mg

^{t}Bu-TAP

363 mg, 80% yield

Cl_4-^{t}Bu-TAP

Chlorination of ^{t}Bu-TAP. **^{t}Bu-TAP** (318 mg, 1.0 mmol) was dissolved in CF_3SO_3H (15 mL). Iodine monochloride (1.6 g, 10 mmol) was added. The reaction mixture was stirred at 80 °C in an oil bath overnight and then allowed to cool to room temperature. The mixture was poured on ice, neutralized with a saturated aqueous solution of Na_2CO_3, and extracted with CH_2Cl_2 (3 × 30 mL). The organic layer was dried over sodium sulfate and filtered. The solvent was removed under reduced pressure, and the residue was purified by column chromatography (silica, hexane/CH_2Cl_2 8:1 v/v) to afford the target product as a light-yellow powder.

Cl_4-^{t}Bu-TAP (363 mg, 80%, a light-yellow powder). ^{1}H NMR (400 MHz, $CDCl_3$) δ 1.71 (s, 18H). ^{13}C NMR (101 MHz, $CDCl_3$) δ 178.30, 150.03, 139.70, 109.92, 41.48, 30.27. HR-MS (ESI, positive): *m/z* $[M + H]^+$ Calcd for $C_{20}H_{19}Cl_4N_4$ 455.0358; Found: 455.0365.

Bromination reaction

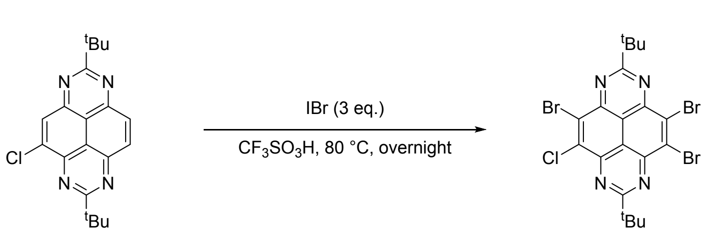

Cl-^{t}Bu-TAP

Br_3Cl-^{t}Bu-TAP, 56% yield

Bromination of Cl-^{t}Bu-TAP. **Cl-^{t}Bu-TAP** (35 mg, 0.1 mmol) was dissolved in CF_3SO_3H (5 mL). Iodine monobromide (62 mg, 0.3 mmol) was added. The reaction mixture was stirred at 80 °C in an oil bath overnight and then allowed to cool to room temperature. The mixture was poured on ice, neutralized with a saturated aqueous solution of Na_2CO_3, and extracted with CH_2Cl_2 (3 × 20 mL). The organic layer was dried over sodium sulfate and filtered. The solvent was removed under reduced pressure, and the residue was purified by column chromatography (silica, hexane/CH_2Cl_2 10:1, v/v) to afford the target product **Br_3Cl-^{t}Bu-TAP** as a light-yellow powder (33 mg, 56%). ^{1}H NMR (400 MHz, $CDCl_3$) δ 1.71–1.70 (m, 18H). ^{13}C NMR (101 MHz, $CDCl_3$) δ 178.55, 178.45, 151.25, 151.09, 150.52, 142.30, 136.58, 136.54, 133.24, 110.73, 110.28, 41.43, 41.42, 30.28. HR-MS (ESI, positive): *m/z* [M + H]$^+$ Calcd for $C_{20}H_{19}Br_3ClN_4$ 586.8843; Found: 586.8836.

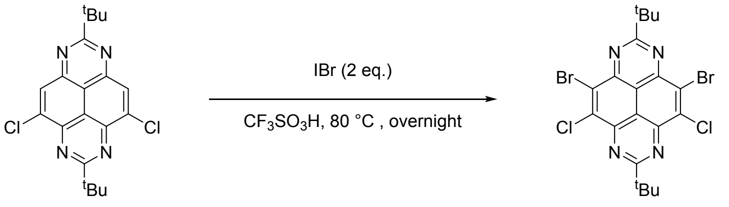

Cl_2-^{t}Bu-TAP **Br_2Cl_2-^{t}Bu-TAP**, 40%

Bromination of Cl_2-^{t}Bu-TAP. Cl_2-^{t}Bu-TAP (35 mg, 0.09 mmol) was dissolved in CF_3SO_3H (5 mL). Iodine monobromide (37 mg, 0.18 mmol) was added. The reaction mixture was stirred at 80 °C in an oil bath overnight and then allowed to cool to room temperature. The mixture was poured on ice, neutralized with a saturated aqueous solution of Na_2CO_3, and extracted with CH_2Cl_2 (3 × 20 mL). The organic layer was dried over sodium sulfate and filtered. The solvent was removed under reduced pressure, and the residue was purified by column chromatography (silica, hexane/CH_2Cl_2 10:1, v/v) to afford the target product **Br_2Cl_2-^{t}Bu-TAP** as a light-yellow powder (22 mg, 40%). ^{1}H NMR (400 MHz, $CDCl_3$) δ 1.71–1.70 (m, 18H). ^{13}C NMR (101 MHz, $CDCl_3$) δ 178.51, 178.32, 150.95, 150.37, 142.41, 133.32, 110.79, 109.89, 41.46, 41.43, 30.29. HR-MS (ESI, positive): *m/z* [M + H]$^+$ Calcd for $C_{20}H_{19}Br_2Cl_2N_4$ 542.9348; Found: 542.9336.

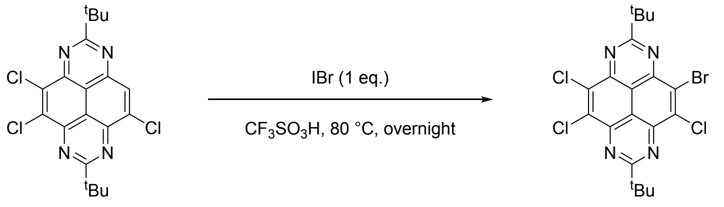

Cl_3-^{t}Bu-TAP **$BrCl_3$-^{t}Bu-TAP**, 45%

Bromination of Cl_3-^{t}Bu-TAP. **Cl_3-^{t}Bu-TAP** (42 mg, 0.1 mmol) was dissolved in CF_3SO_3H (10 mL). Iodine monobromide (21 mg, 0.1 mmol) was added. The reaction mixture was stirred at 80 °C in an oil bath overnight and then allowed to cool to room temperature. The mixture was poured on ice, neutralized with a saturated aqueous solution of Na_2CO_3, and extracted with CH_2Cl_2 (3 × 20 mL). The organic layer was dried over sodium sulfate and filtered. The solvent was removed under reduced pressure, and the residue was purified by column chromatography (silica, hexane/CH_2Cl_2 10:1, v/v) to afford the target product **$BrCl_3$-^{t}Bu-TAP** as a light-yellow powder (23 mg, 45%). ^{1}H NMR (400 MHz, $CDCl_3$) δ 1.71–1.70 (m, 18H). ^{13}C NMR (101 MHz, $CDCl_3$) δ 178.41, 178.31, 150.82, 150.23, 150.17, 142.48, 139.63, 139.60, 133.43, 110.36, 109.91, 41.47, 41.45, 30.28. HR-MS (ESI, positive): *m/z* [M + H]$^+$ Calcd for $C_{20}H_{19}BrCl_3N_4$ 498.9853; Found: 498.9841.

X-Ray Diffraction Analysis of 4,10-Cl_2-^{t}Bu-TAP

Approaches to single-crystal growth: To grow single crystals of **4,10-Cl_2-tBu-TAP**, 10 mg of the sample was dissolved in 2 mL of dichloromethane in a small vial. The solution was then left to evaporate slowly at ambient temperature for 24 hours.

Crystal-Structure Determination. A crystal of $C_{20}H_{20}Cl_2N_4$ immersed in parabar oil was mounted at ambient conditions and transferred into the stream of nitrogen (173 K). All measurements were made on a *RIGAKU Synergy S* area-detector diffractometer[2] using mirror optics monochromated Cu *K*α radiation (λ = 1.54184 Å). The unit cell constants and an orientation matrix for data collection were obtained from a least-squares refinement of the setting angles of reflections in the range 3.405° < θ < 80.145°. A total of 11236 frames were collected using ω scans, with 0.15 seconds exposure time (0.5 s for high-angle reflections), a rotation angle of 0.5° per frame, a crystal-detector distance of 31.0 mm, at T = 173.00(10) K.

Data reduction was performed using the *CrysAlisPro*[2] program. The intensities were corrected for Lorentz and polarization effects, and a numerical absorption correction based on gaussian integration over a multifaceted crystal model with additional empirical absorption correction using spherical harmonics using SCALE3 ABSPACK in *CrysAlisPro*[2] was applied. Data collection and refinement parameters are given in Table S1.

The structure was solved by intrinsic phasing using *SHELXT*[3], which revealed the positions of all non-hydrogen atoms of the title compound. All non-hydrogen atoms were refined anisotropically. H-atoms were assigned in geometrically calculated positions and refined using a riding model where each H-atom was assigned a fixed isotropic displacement parameter with a value equal to 1.2Ueq of its parent atom, except for those attached to methyl groups, where H atoms were located from the difference density map and had their positions and isotropic displacement parameters refined freely.

Refinement of the structure was carried out on F^2 using full-matrix least-squares procedures, which minimized the function $\Sigma w(F_o^2 - F_c^2)^2$. The weighting scheme was based on counting statistics and included a factor to downweight the intense reflections. All calculations were performed using the *SHELXL-2014/7*[4] program in OLEX2.[5]

Disorder model was used for parts of the structure where the occupancies of each disorder component was refined through the use of a free variable. The sum of equivalent components was constrained to 1, i.e. 100%. Final occupancies can be seen on Table S9.

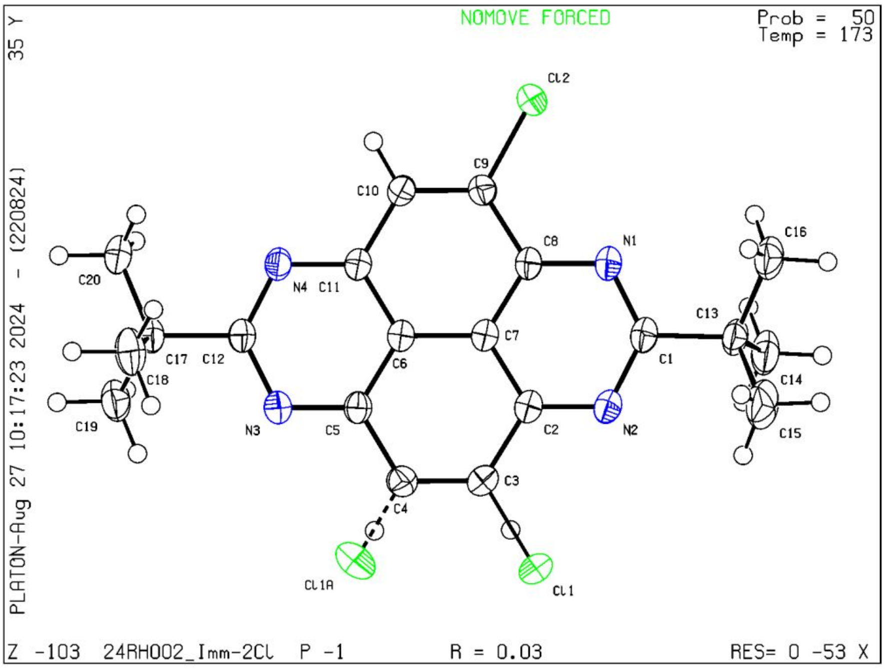

Figure S1 ORTEP view of **4,10-Cl$_2$-^{t}Bu-TAP** showing the atomic labeling scheme. Thermal ellipsoids drawn at 50% probability levels.

Table S2 Crystal data and structure refinement for 4,10-Cl_2-^{t}Bu-TAP.

Identification code	24RH002_Imm-2Cl
Empirical formula	$C_{20}H_{20}Cl_2N_4$
Formula weight	387.30
Temperature/K	173.00(10)
Crystal system	triclinic
Space group	P-1
a/Å	5.94797(7)
b/Å	12.23597(10)
c/Å	14.26402(11)
α/°	67.7619(8)
β/°	78.1599(9)
γ/°	87.1751(8)
Volume/Å^3	940.002(16)
Z	2
ρ_{calc}g/cm^3	1.368
μ/mm^{-1}	3.188
F(000)	404.0
Crystal size/mm^3	0.619 × 0.326 × 0.169
Radiation	Cu Kα (λ = 1.54184)
2Θ range for data collection/°	6.836 to 148.948
Index ranges	$-7 \leq h \leq 7$, $-15 \leq k \leq 15$, $-17 \leq l \leq 17$
Reflections collected	37802
Independent reflections	3829 [R_{int} = 0.0233, R_{sigma} = 0.0085]
Data/restraints/parameters	3829/1/318
Goodness-of-fit on F^2	1.035
Final R indexes [I>=2σ (I)]	R_1 = 0.0286, wR_2 = 0.0795
Final R indexes [all data]	R_1 = 0.0288, wR_2 = 0.0796
Largest diff. peak/hole / e Å^{-3}	0.30/-0.27

Table S3 Fractional Atomic Coordinates (×10^4) and Equivalent Isotropic Displacement Parameters (Å^2×10^3) for 4,10-Cl$_2$-^{t}Bu-TAP. U$_{eq}$ is defined as 1/3 of of the trace of the orthogonalised U$_{IJ}$ tensor.

Atom	*x*	*y*	*z*	U(eq)
Cl2	12671.7(5)	3364.1(2)	6831.2(2)	31.26(10)
Cl1	1313.7(5)	6682.3(3)	7835.3(2)	35.18(12)
N3	7703.7(18)	8484.1(9)	4656.7(8)	27.5(2)
N4	11190.7(18)	7467.3(9)	4380.0(8)	27.9(2)
N1	8033.7(18)	3685.9(9)	8039.5(8)	27.8(2)
N2	4580.3(18)	4718.8(9)	8328.4(8)	28.5(2)
C6	8223(2)	6546.8(10)	5859.8(9)	24.8(2)
C2	5307(2)	5605.6(11)	7427.9(9)	26.0(2)
C5	6911(2)	7558.8(10)	5533.1(9)	25.6(2)
C7	7429(2)	5583.1(10)	6787.5(9)	24.8(2)
C8	8759(2)	4578.2(10)	7134.0(9)	25.2(2)
C11	10387(2)	6540.7(10)	5251.6(9)	25.5(2)
C12	9802(2)	8388.7(10)	4123.1(9)	26.3(2)
C4	4706(2)	7582.3(11)	6173.9(10)	28.6(3)
C9	10974(2)	4580.9(10)	6471.7(9)	26.0(2)
C1	5989(2)	3803.1(11)	8596.8(9)	28.7(3)
C3	3955(2)	6655.3(11)	7069.5(9)	27.4(2)
C10	11755(2)	5514.7(11)	5582.7(9)	27.3(2)
C17	10748(2)	9457.9(11)	3152.1(9)	29.6(3)
C13	5103(2)	2798.0(12)	9632.2(9)	33.2(3)
C19	8858(3)	10005.0(14)	2548.7(12)	40.8(3)
C20	12705(3)	9105.8(14)	2457.9(12)	44.1(4)
C14	3390(3)	2041.1(15)	9446.6(12)	44.6(4)
C18	11635(3)	10354.3(13)	3518.1(12)	43.2(4)
C16	7055(3)	2026.4(17)	10038.4(13)	51.2(4)
C15	3889(4)	3305.6(18)	10421.9(13)	60.9(5)
Cl1A	3270(40)	8701(17)	5837(16)	62(6)

Table S4 Anisotropic Displacement Parameters (Å^2×10^3) for 4,10-Cl$_2$-^{t}Bu-TAP. The Anisotropic displacement factor exponent takes the form: -2π^2[h^{2}a*^{2}U$_{11}$+2hka*b*U$_{12}$+…].

Atom	U_{11}	U_{22}	U_{33}	U_{23}	U_{13}	U_{12}
Cl2	32.52(17)	27.75(16)	28.45(16)	-5.51(12)	-6.06(11)	4.73(11)
Cl1	26.71(17)	39.00(19)	33.81(18)	-11.87(14)	3.17(12)	1.68(12)
N3	30.5(5)	24.1(5)	25.2(5)	-6.4(4)	-4.9(4)	-2.0(4)
N4	27.8(5)	27.0(5)	23.2(5)	-4.1(4)	-2.8(4)	-2.7(4)
N1	30.3(5)	27.5(5)	21.3(5)	-4.9(4)	-3.5(4)	-3.7(4)
N2	28.0(5)	31.5(5)	22.3(5)	-7.1(4)	-1.7(4)	-3.9(4)
C6	26.5(6)	24.7(6)	21.9(5)	-7.3(5)	-4.2(4)	-2.6(4)
C2	27.0(6)	27.8(6)	22.4(5)	-8.5(5)	-4.0(4)	-4.1(5)
C5	27.9(6)	24.2(5)	24.0(6)	-8.2(5)	-4.9(5)	-2.8(4)
C7	26.3(6)	25.3(6)	21.8(5)	-7.7(5)	-3.8(4)	-3.3(4)
C8	27.9(6)	25.3(6)	20.8(5)	-6.4(4)	-4.7(4)	-3.2(4)
C11	26.0(6)	26.1(6)	22.0(5)	-6.4(5)	-3.9(4)	-3.2(4)
C12	29.2(6)	24.3(6)	23.6(6)	-6.4(5)	-5.2(5)	-4.3(4)
C4	28.8(6)	26.9(6)	29.7(6)	-10.2(5)	-5.7(5)	1.1(5)
C9	27.5(6)	25.2(6)	24.1(6)	-7.5(5)	-6.0(5)	0.8(4)
C1	30.7(6)	30.6(6)	21.3(6)	-5.8(5)	-4.2(5)	-5.3(5)
C3	24.3(6)	30.9(6)	27.3(6)	-12.6(5)	-2.0(5)	-1.7(5)
C10	25.2(6)	28.8(6)	24.1(6)	-7.2(5)	-2.0(4)	-0.4(5)
C17	32.5(6)	24.7(6)	25.2(6)	-2.1(5)	-4.4(5)	-4.9(5)
C13	31.9(6)	36.6(7)	20.5(6)	-0.6(5)	-0.9(5)	-4.1(5)
C19	40.8(8)	37.3(7)	32.7(7)	2.4(6)	-11.3(6)	-5.1(6)
C20	46.3(9)	34.8(7)	32.8(7)	0.3(6)	7.1(6)	-2.5(6)
C14	39.2(8)	45.4(8)	32.8(7)	3.9(6)	-4.5(6)	-13.5(6)
C18	56.2(9)	31.0(7)	37.2(8)	-4.3(6)	-11.0(7)	-15.1(7)
C16	35.7(8)	60.2(10)	32.6(8)	10.8(7)	-6.5(6)	-3.9(7)
C15	87.3(15)	54.8(10)	24.3(7)	-6.5(7)	7.8(8)	2.0(10)
Cl1A	60(12)	45(8)	60(12)	-1(8)	-10(9)	23(7)

Table S5 Bond Lengths for 4,10-Cl$_2$-^{t}Bu-TAP.

Atom	Atom	Length/Å	Atom	Atom	Length/Å
Cl2	C9	1.7280(12)	C5	C4	1.4427(17)
Cl1	C3	1.7266(12)	C7	C8	1.4093(17)
N3	C5	1.3439(16)	C8	C9	1.4545(17)
N3	C12	1.3469(16)	C11	C10	1.4383(17)
N4	C11	1.3424(15)	C12	C17	1.5257(16)
N4	C12	1.3443(16)	C4	C3	1.3553(17)
N1	C8	1.3404(15)	C4	Cl1A	1.545(17)
N1	C1	1.3431(17)	C9	C10	1.3576(17)
N2	C2	1.3331(16)	C1	C13	1.5299(16)
N2	C1	1.3486(17)	C17	C19	1.5297(19)
C6	C5	1.4060(17)	C17	C20	1.524(2)
C6	C7	1.4077(16)	C17	C18	1.5352(19)
C6	C11	1.3982(18)	C13	C14	1.535(2)
C2	C7	1.4042(17)	C13	C16	1.526(2)
C2	C3	1.4584(17)	C13	C15	1.526(2)

Table S6 Bond Angles for 4,10-Cl$_2$-^{t}Bu-TAP.

Atom	Atom	Atom	Angle/°	Atom	Atom	Atom	Angle/°
C5	N3	C12	116.58(10)	C5	C4	Cl1A	118.6(8)
C11	N4	C12	116.55(11)	C3	C4	C5	120.32(11)
C8	N1	C1	116.60(11)	C3	C4	Cl1A	121.1(8)
C2	N2	C1	116.76(11)	C8	C9	Cl2	119.05(9)
C5	C6	C7	121.37(11)	C10	C9	Cl2	118.99(10)
C11	C6	C5	117.90(11)	C10	C9	C8	121.95(11)
C11	C6	C7	120.69(11)	N1	C1	N2	126.50(11)
N2	C2	C7	121.58(11)	N1	C1	C13	117.98(11)
N2	C2	C3	121.63(11)	N2	C1	C13	115.50(11)
C7	C2	C3	116.77(11)	C2	C3	Cl1	117.31(9)
N3	C5	C6	120.94(11)	C4	C3	Cl1	120.32(10)
N3	C5	C4	120.98(11)	C4	C3	C2	122.37(11)
C6	C5	C4	118.07(11)	C9	C10	C11	120.75(11)
C6	C7	C8	121.74(11)	C12	C17	C19	110.69(11)
C2	C7	C6	121.07(11)	C12	C17	C18	106.54(10)
C2	C7	C8	117.14(11)	C19	C17	C18	109.66(12)
N1	C8	C7	121.40(11)	C20	C17	C12	110.56(11)
N1	C8	C9	122.17(11)	C20	C17	C19	109.30(12)
C7	C8	C9	116.41(11)	C20	C17	C18	110.06(13)
N4	C11	C6	121.29(11)	C1	C13	C14	107.25(10)
N4	C11	C10	120.29(11)	C16	C13	C1	111.38(11)
C6	C11	C10	118.42(11)	C16	C13	C14	108.94(13)
N3	C12	C17	116.68(11)	C16	C13	C15	109.76(14)
N4	C12	N3	126.73(11)	C15	C13	C1	109.79(12)
N4	C12	C17	116.53(11)	C15	C13	C14	109.68(14)

Table S7 Torsion Angles for 4,10-Cl_2-^{t}Bu-TAP.

A	B	C	D	Angle/°	A	B	C	D	Angle/°
Cl2	C9	C10	C11	179.51(9)	C5	C6	C7	C8	-178.35(11)
N3	C5	C4	C3	-178.25(11)	C5	C6	C11	N4	-0.28(18)
N3	C5	C4	Cl1A	-0.5(11)	C5	C6	C11	C10	179.44(10)
N3	C12	C17	C19	-40.71(15)	C5	C4	C3	Cl1	-179.88(9)
N3	C12	C17	C20	-161.97(12)	C5	C4	C3	C2	0.04(19)
N3	C12	C17	C18	78.45(14)	C7	C6	C5	N3	178.65(10)
N4	C11	C10	C9	178.91(11)	C7	C6	C5	C4	-0.40(17)
N4	C12	C17	C19	141.91(12)	C7	C6	C11	N4	-178.15(10)
N4	C12	C17	C20	20.65(16)	C7	C6	C11	C10	1.57(18)
N4	C12	C17	C18	-98.93(14)	C7	C2	C3	Cl1	178.66(9)
N1	C8	C9	Cl2	3.17(16)	C7	C2	C3	C4	-1.27(17)
N1	C8	C9	C10	-176.35(11)	C7	C8	C9	Cl2	-178.54(8)
N1	C1	C13	C14	-98.76(14)	C7	C8	C9	C10	1.94(17)
N1	C1	C13	C16	20.36(18)	C8	N1	C1	N2	1.16(18)
N1	C1	C13	C15	142.14(15)	C8	N1	C1	C13	179.70(10)
N2	C2	C7	C6	-177.21(11)	C8	C9	C10	C11	-0.97(19)
N2	C2	C7	C8	0.37(17)	C11	N4	C12	N3	0.95(18)
N2	C2	C3	Cl1	-2.47(16)	C11	N4	C12	C17	178.03(10)
N2	C2	C3	C4	177.61(11)	C11	C6	C5	N3	0.80(17)
N2	C1	C13	C14	79.95(15)	C11	C6	C5	C4	-178.25(11)
N2	C1	C13	C16	-160.94(13)	C11	C6	C7	C2	176.92(11)
N2	C1	C13	C15	-39.16(18)	C11	C6	C7	C8	-0.56(18)
C6	C5	C4	C3	0.80(18)	C12	N3	C5	C6	-0.45(17)
C6	C5	C4	Cl1A	178.6(11)	C12	N3	C5	C4	178.57(11)
C6	C7	C8	N1	177.14(11)	C12	N4	C11	C6	-0.53(17)
C6	C7	C8	C9	-1.17(17)	C12	N4	C11	C10	179.75(11)
C6	C11	C10	C9	-0.82(18)	C1	N1	C8	C7	-0.27(17)
C2	N2	C1	N1	-1.22(19)	C1	N1	C8	C9	177.94(11)
C2	N2	C1	C13	-179.79(10)	C1	N2	C2	C7	0.38(17)

Table S7 Torsion Angles for 4,10-Cl_2-^{t}Bu-TAP.

A	B	C	D	Angle/°	A	B	C	D	Angle/°
C2	C7	C8	N1	-0.43(17)	C1	N2	C2	C3	-178.44(10)
C2	C7	C8	C9	-178.74(10)	C3	C2	C7	C6	1.66(17)
C5	N3	C12	N4	-0.46(18)	C3	C2	C7	C8	179.25(10)
C5	N3	C12	C17	-177.53(10)	Cl1A	C4	C3	C2	-177.7(11)
C5	C6	C7	C2	-0.87(18)					

Table S8 Hydrogen Atom Coordinates (Å×10^4) and Isotropic Displacement Parameters (Å^2×10^3) for 4,10-Cl$_2$-^{t}Bu-TAP.

**Atom**	***x***	***y***	***z***	**U(eq)**
H4	3774.72	8250.89	5967.21	34
H3	2496.88	6690.56	7478.51	33
H10	13220.81	5490.21	5174.77	33
H18A	12830(30)	9987(16)	3896(14)	47(5)
H19A	9430(30)	10712(17)	1925(15)	53(5)
H14A	2150(40)	2509(18)	9157(16)	61(6)
H19B	8340(30)	9469(17)	2287(15)	54(5)
H14B	2800(30)	1421(17)	10099(16)	54(5)
H18B	10340(30)	10606(16)	3961(15)	51(5)
H20A	13280(30)	9800(18)	1813(16)	60(5)
H20B	12180(30)	8462(19)	2261(16)	61(6)
H18C	12280(30)	11059(17)	2913(15)	51(5)
H19C	7510(40)	10205(18)	2970(16)	60(6)
H15A	5020(40)	3820(20)	10528(18)	77(7)
H16A	8180(40)	2548(19)	10168(16)	67(6)
H20C	13950(30)	8832(17)	2809(15)	52(5)
H16B	7760(40)	1639(19)	9536(18)	69(6)
H14C	4180(40)	1669(18)	8920(17)	66(6)
H16C	6450(40)	1408(19)	10718(18)	66(6)
H15B	2580(40)	3757(19)	10174(17)	67(6)
H15C	3410(40)	2637(19)	11074(17)	63(6)

Table S9 Atomic occupancy for 4,10-Cl$_2$-^{t}Bu-TAP.

**Atom**	***Occupancy***	**Atom**	***Occupancy***	**Atom**	***Occupancy***
Cl1	0.9776(13)	H4	0.9776(13)	H3	0.0224(13)
Cl1A	0.0224(13)				

CV and DPV Spectra

Cyclic voltammetry (CV) and differential pulse voltammetry (DPV) measurements were performed using a typical one-compartment, three-electrode setup driven by a Metrohm PGSTAT101 potentiostat. A platinum (Pt) disk (diameter = 3 mm, circular surface area = 7.0686 mm^2) was used as the working electrode, a glassy carbon rod as the counter electrode, and an Ag/AgCl electrode (2 M LiCl in ethanol) as the reference electrode. The electrochemical experiments were carried out under Argon atmosphere at room temperature, and 0.1 M CH_2Cl_2 solution of tetrabutylammonium hexafluorophosphate ($TBAPF_6$) was used as a supporting electrolyte at a scan rate of 100 mV/s. CV plotting followed an IUPAC convention.

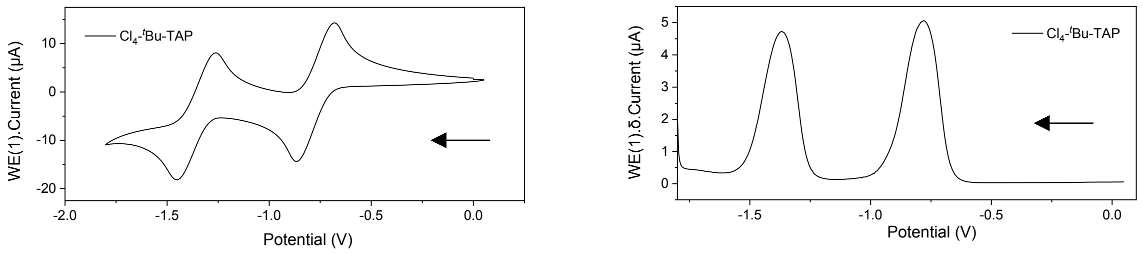

Figures S2 CV and DPV spectra of **Cl_4-^{t}Bu-TAP** (0.02 mM). A platinum (Pt) disk was used as the working electrode, a glassy carbon rod as the counter electrode, and an Ag/AgCl electrode (2 M LiCl in ethanol) as the reference electrode. 0.1 M CH_2Cl_2 solution of $TBAPF_6$ was used as a supporting electrolyte at a scan rate of 100 mV/s. The potential was swept in the negative direction, starting from 0 V.

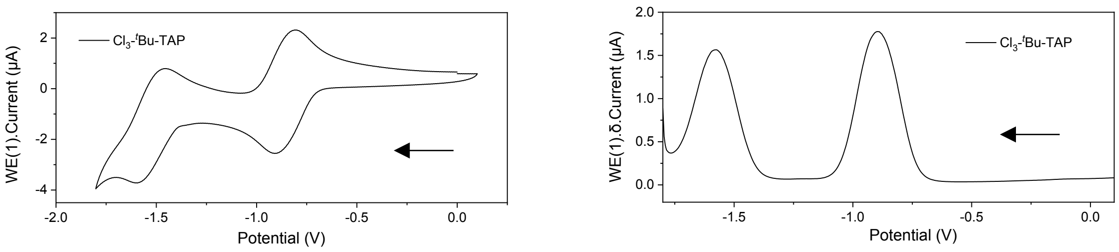

Figures S3 CV and DPV spectra of **Cl_3-^{t}Bu-TAP** (0.02 mM). A platinum (Pt) disk was used as the working electrode, a glassy carbon rod as the counter electrode, and an Ag/AgCl electrode (2 M LiCl in ethanol) as the reference electrode. 0.1 M CH_2Cl_2 solution of $TBAPF_6$ was used as a supporting electrolyte at a scan rate of 100 mV/s. The potential was swept in the negative direction, starting from 0 V.

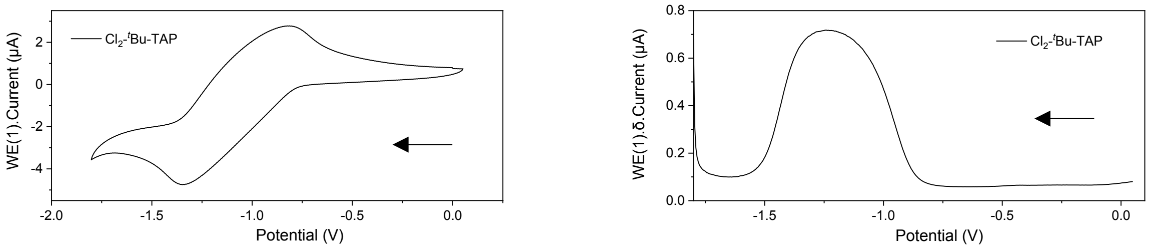

Figures S4 CV and DPV spectra of **4,10-Cl$_2$-^{t}Bu-TAP** (0.02 mM). A platinum (Pt) disk was used as the working electrode, a glassy carbon rod as the counter electrode, and an Ag/AgCl electrode (2 M LiCl in ethanol) as the reference electrode. 0.1 M CH_2Cl_2 solution of $TBAPF_6$ was used as a supporting electrolyte at a scan rate of 100 mV/s. The potential was swept in the negative direction, starting from 0 V.

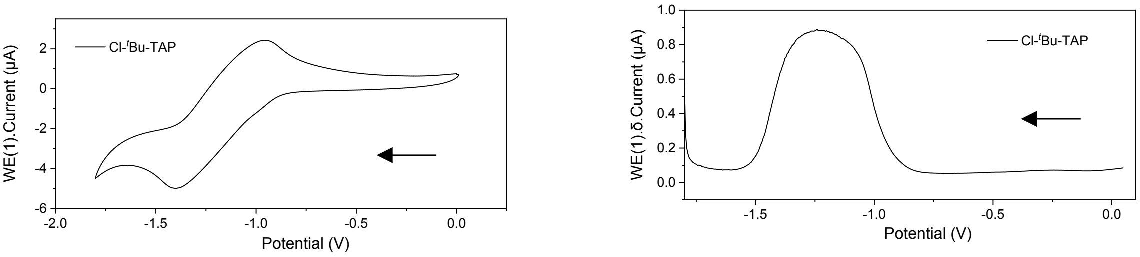

Figures S5 CV and DPV spectra of **Cl-^{t}Bu-TAP** (0.02 mM). A platinum (Pt) disk was used as the working electrode, a glassy carbon rod as the counter electrode, and an Ag/AgCl electrode (2 M LiCl in ethanol) as the reference electrode. 0.1 M CH_2Cl_2 solution of $TBAPF_6$ was used as a supporting electrolyte at a scan rate of 100 mV/s. The potential was swept in the negative direction, starting from 0 V.

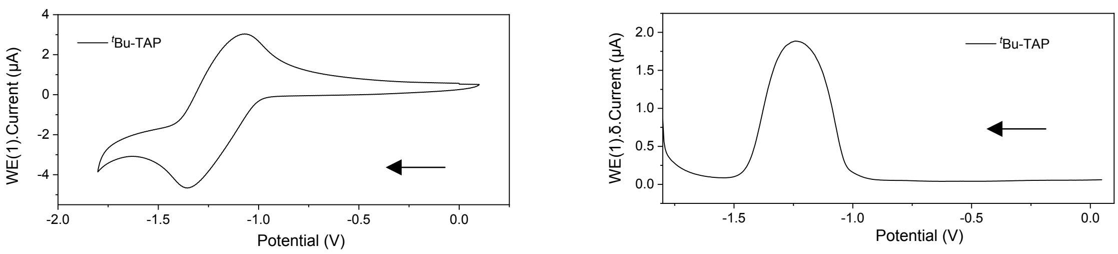

Figures S6 CV and DPV spectra of **^{t}Bu-TAP** (0.02 mM). A platinum (Pt) disk was used as the working electrode, a glassy carbon rod as the counter electrode, and an Ag/AgCl electrode (2 M LiCl in ethanol) as the reference electrode. 0.1 M CH_2Cl_2 solution of $TBAPF_6$ was used as a supporting electrolyte at a scan rate of 100 mV/s. The potential was swept in the negative direction, starting from 0 V.

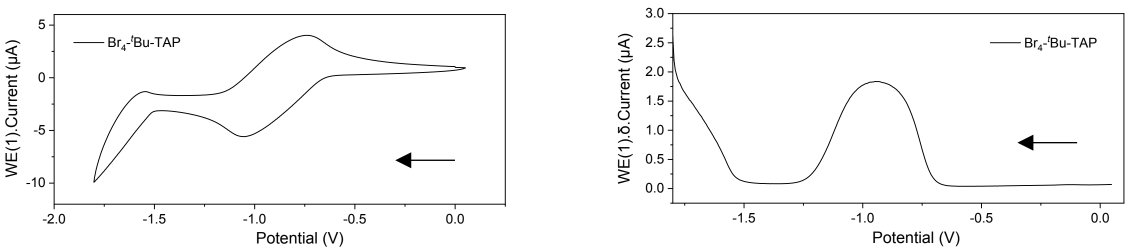

Figures S7 CV and DPV spectra of **Br_4-^{t}Bu-TAP** (0.02 mM). A platinum (Pt) disk was used as the working electrode, a glassy carbon rod as the counter electrode, and an Ag/AgCl electrode (2 M LiCl in ethanol) as the reference electrode. 0.1 M CH_2Cl_2 solution of $TBAPF_6$ was used as a supporting electrolyte at a scan rate of 100 mV/s. The potential was swept in the negative direction, starting from 0 V.

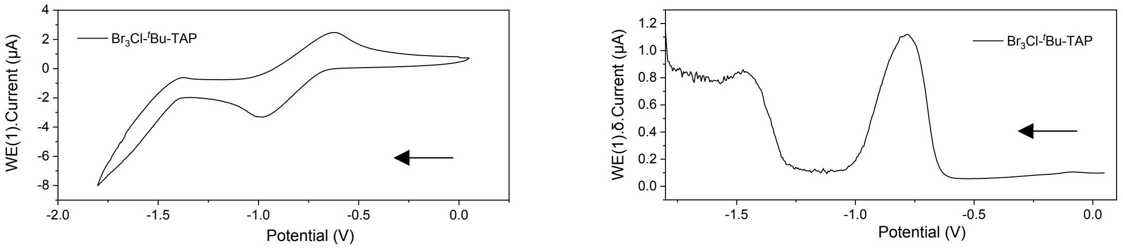

Figures S8 CV and DPV spectra of **Br_3Cl-^{t}Bu-TAP** (0.02 mM). A platinum (Pt) disk was used as the working electrode, a glassy carbon rod as the counter electrode, and an Ag/AgCl electrode (2 M LiCl in ethanol) as the reference electrode. 0.1 M CH_2Cl_2 solution of $TBAPF_6$ was used as a supporting electrolyte at a scan rate of 100 mV/s. The potential was swept in the negative direction, starting from 0 V.

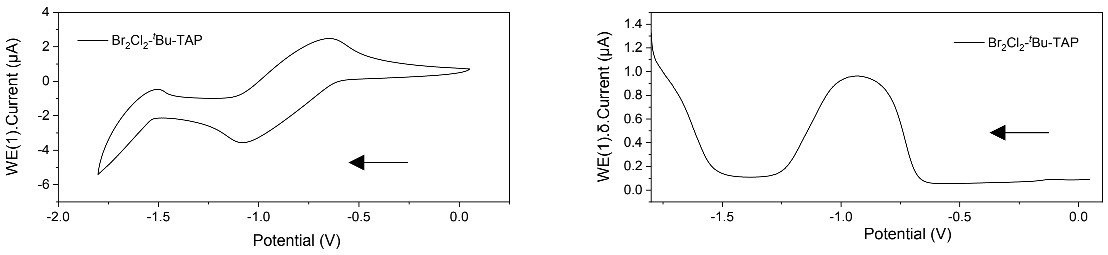

Figures S9 CV and DPV spectra of **Br_2Cl_2-^{t}Bu-TAP** (0.02 mM). A platinum (Pt) disk was used as the working electrode, a glassy carbon rod as the counter electrode, and an Ag/AgCl electrode (2 M LiCl in ethanol) as the reference electrode. 0.1 M CH_2Cl_2 solution of $TBAPF_6$ was used as a supporting electrolyte at a scan rate of 100 mV/s. The potential was swept in the negative direction, starting from 0 V.

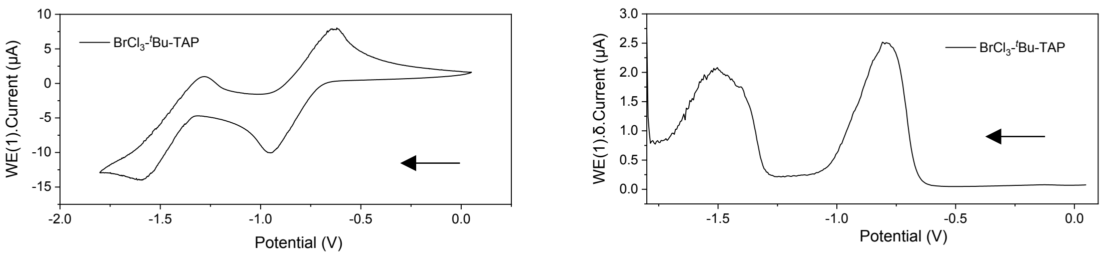

Figures S10 CV and DPV spectra of **BrCl$_3$-^{t}Bu-TAP** (0.02 mM). A platinum (Pt) disk was used as the working electrode, a glassy carbon rod as the counter electrode, and an Ag/AgCl electrode (2 M LiCl in ethanol) as the reference electrode. 0.1 M CH_2Cl_2 solution of $TBAPF_6$ was used as a supporting electrolyte at a scan rate of 100 mV/s. The potential was swept in the negative direction, starting from 0 V.

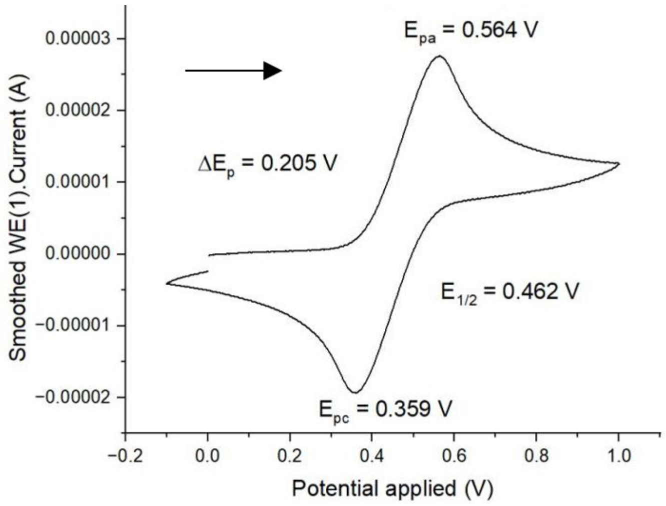

Figures S11 CV spectrum of ferrocene under the same conditions. A platinum (Pt) disk was used as the working electrode, a glassy carbon rod as the counter electrode, and an Ag/AgCl electrode (2 M LiCl in ethanol) as the reference electrode. 0.1 M CH_2Cl_2 solution of $TBAPF_6$ was used as a supporting electrolyte at a scan rate of 100 mV/s. The potential was swept in the positive direction, starting from 0 V.

NMR and MS Spectra

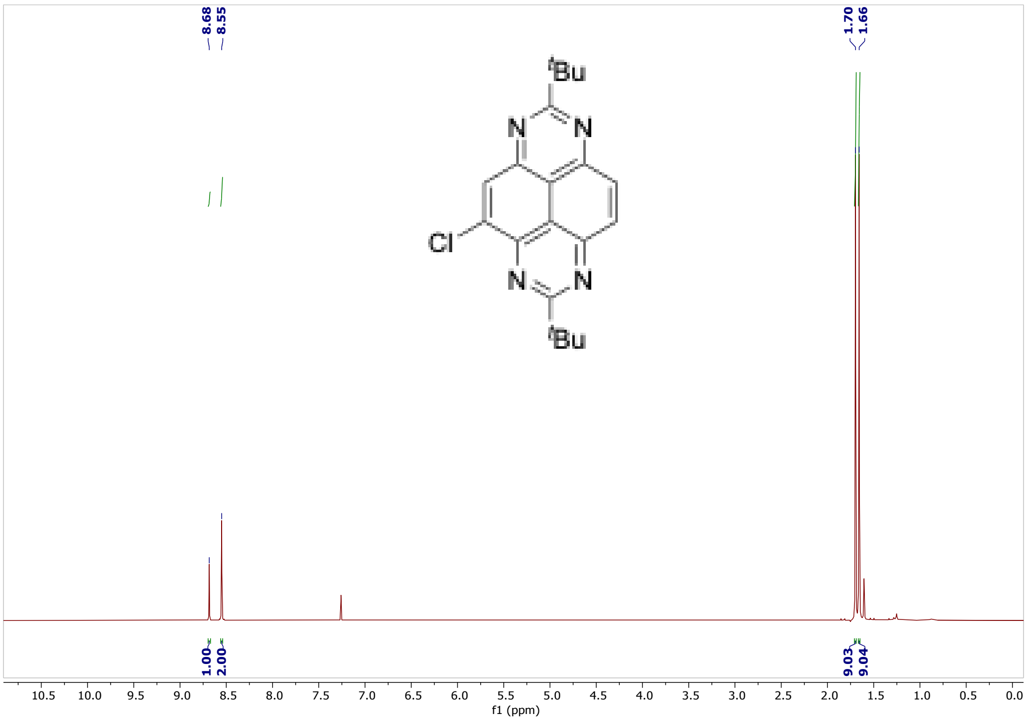

^{1}H NMR spectrum (400 MHz) of **Cl-^{t}Bu-TAP** in $CDCl_3$.

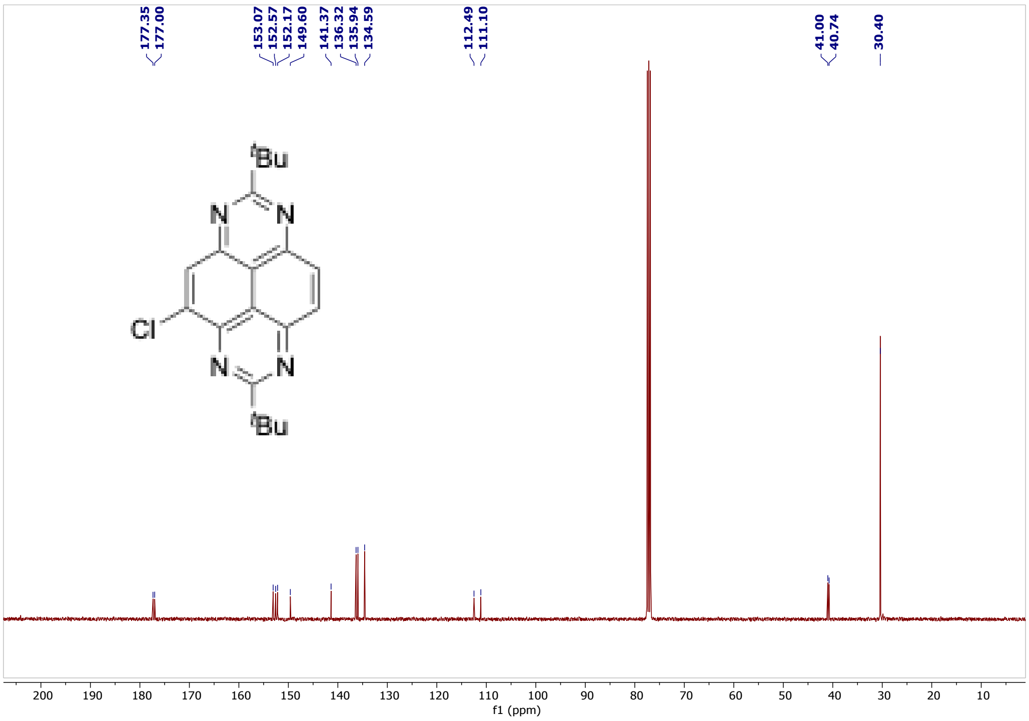

^{13}C NMR spectrum (101 MHz) of **Cl-^{t}Bu-TAP** in $CDCl_3$.

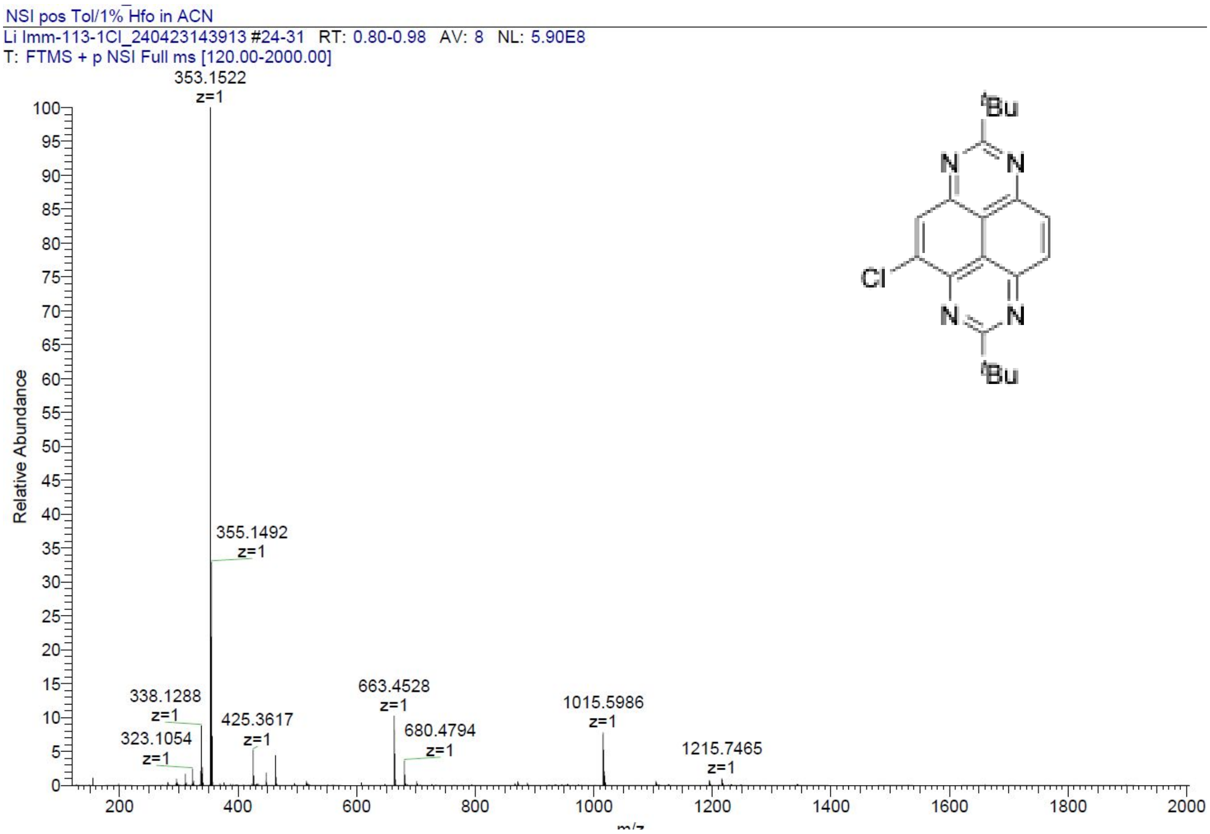

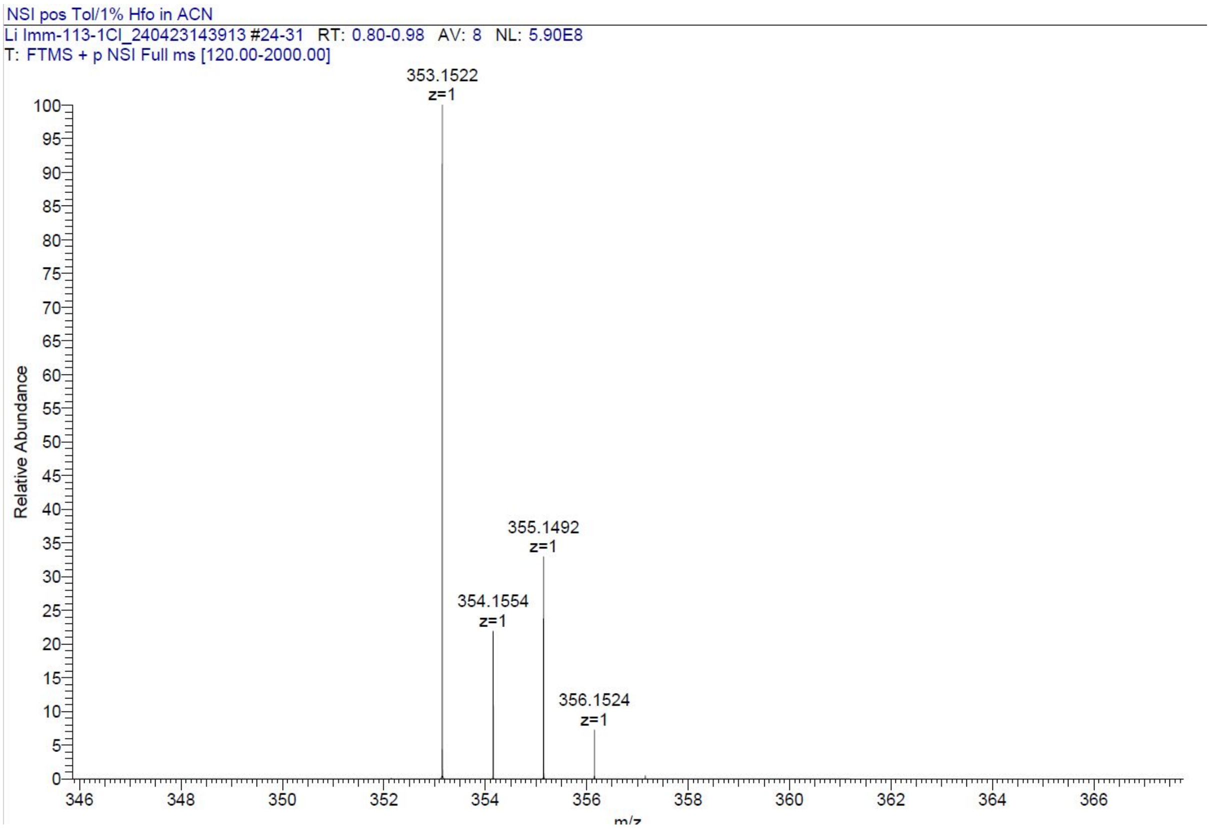

HR-MS spectrum of **Cl-^{t}Bu-TAP**.

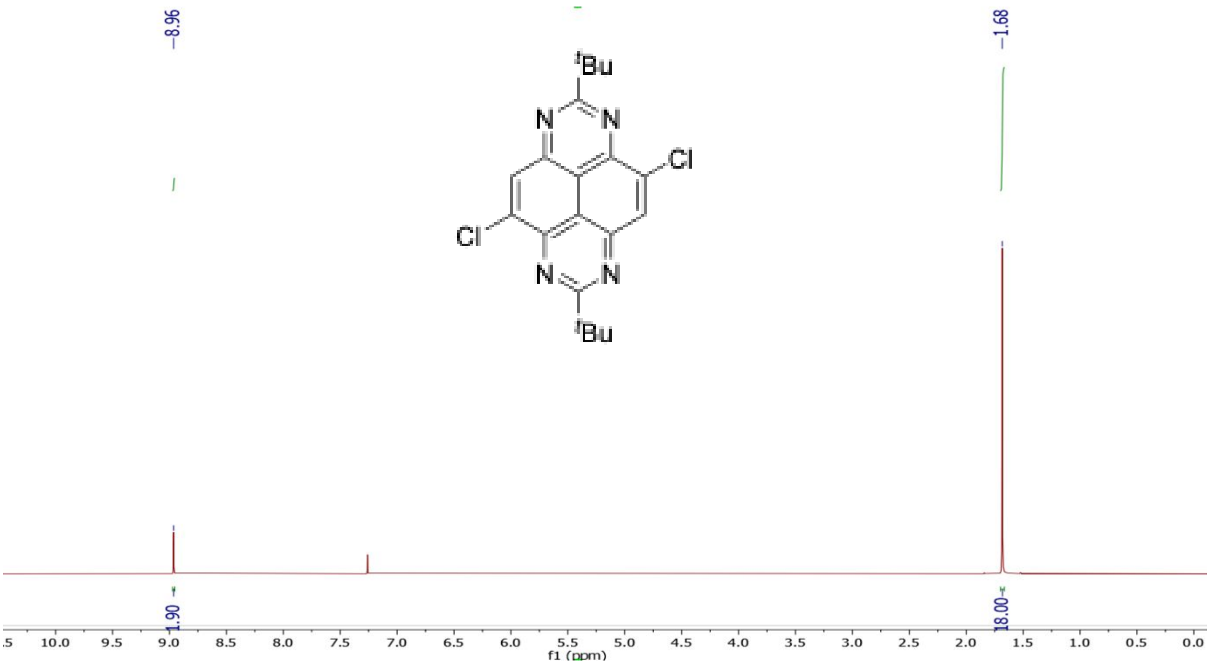

^{1}H NMR spectrum (400 MHz) of **4,9-Cl_2-*t*Bu-TAP** in $CDCl_3$.

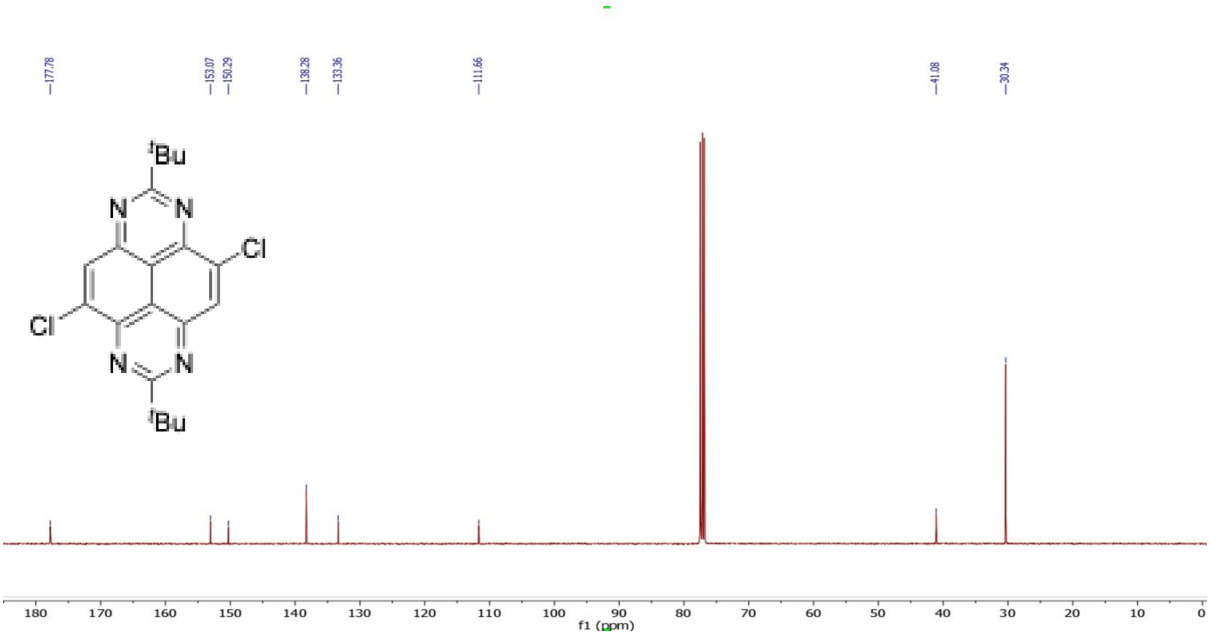

^{13}C NMR spectrum (101 MHz) of **4,9-Cl_2-*t*Bu-TAP** in $CDCl_3$.

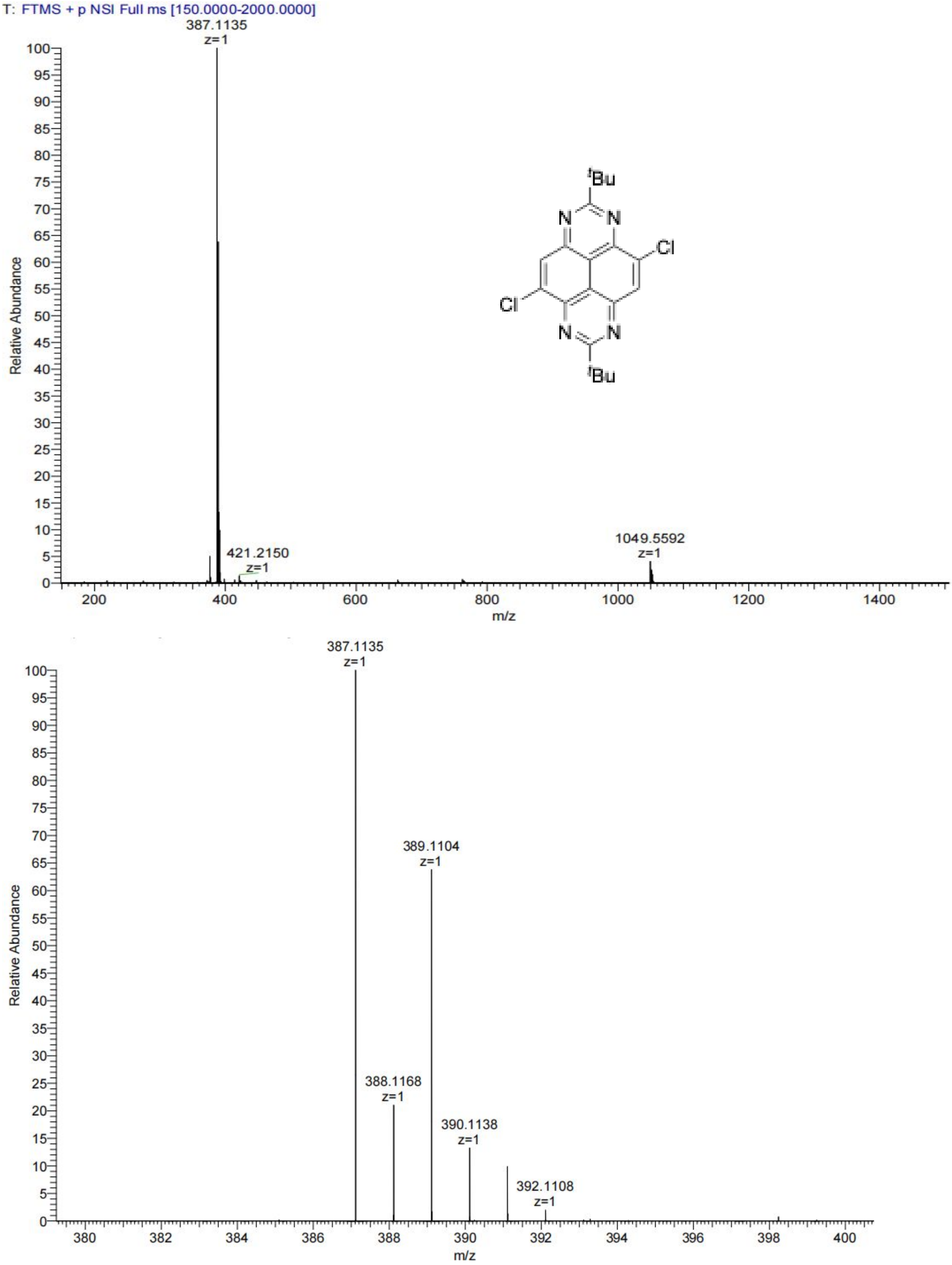

HR-MS spectrum of **4,9-Cl$_2$-^{t}Bu-TAP**.

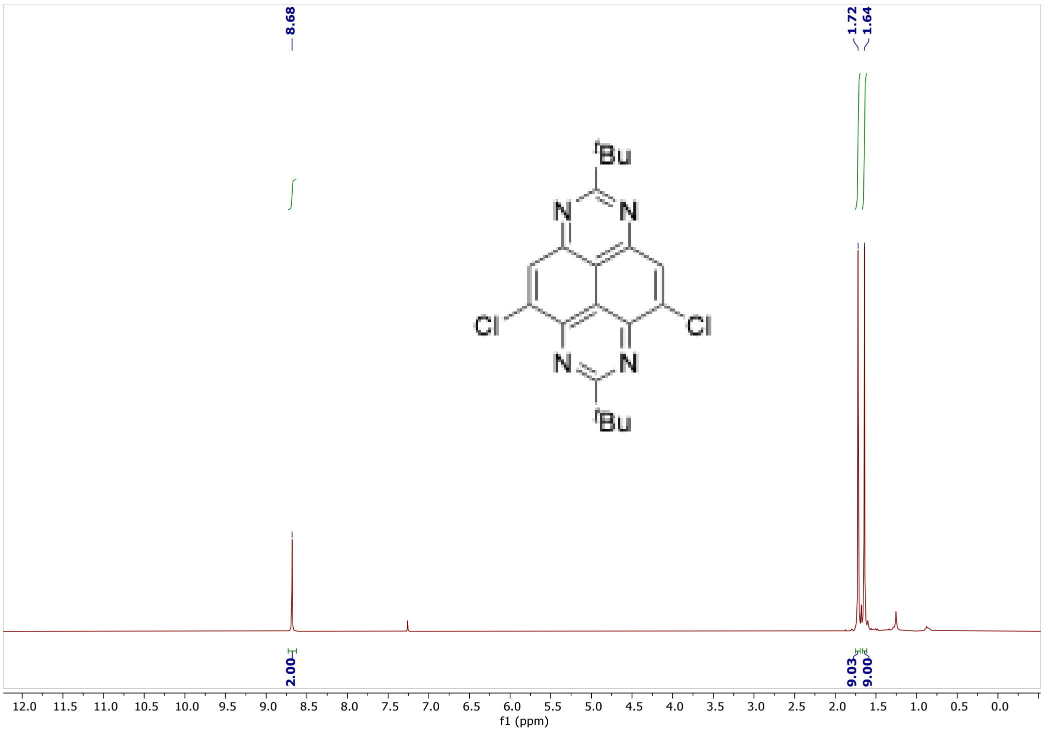

^{1}H NMR spectrum (400 MHz) of **4,10-Cl$_2$-^{t}Bu-TAP** in $CDCl_3$.

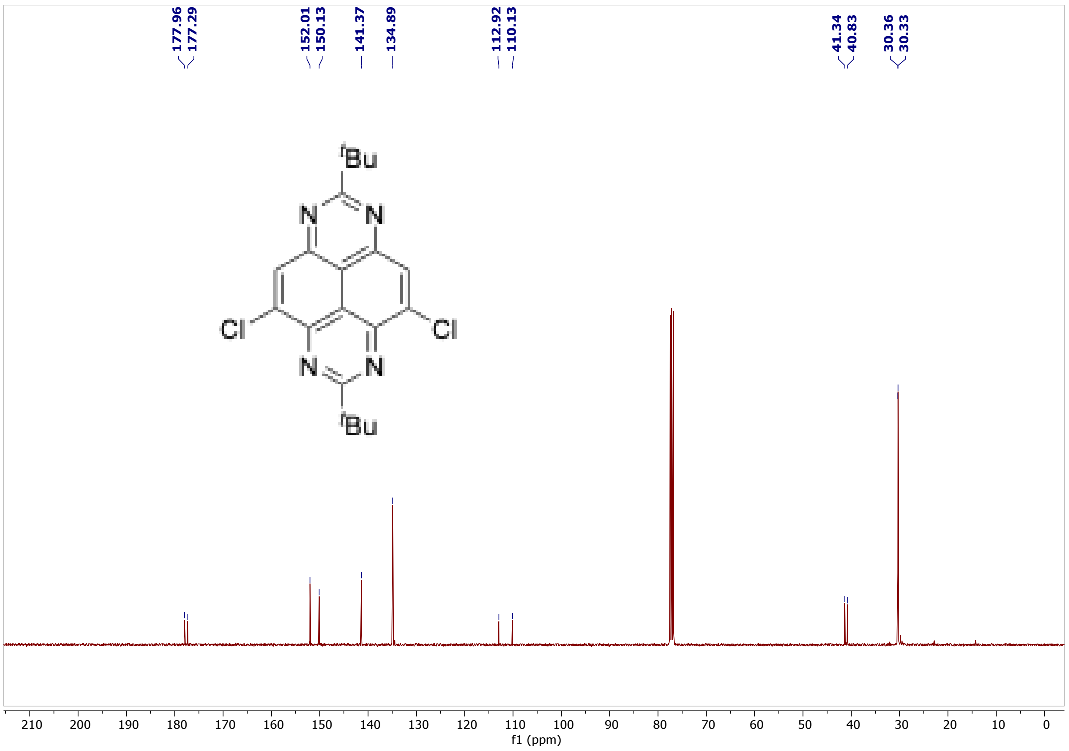

^{13}C NMR spectrum (101 MHz) of **4,10-Cl$_2$-^{t}Bu-TAP** in $CDCl_3$.

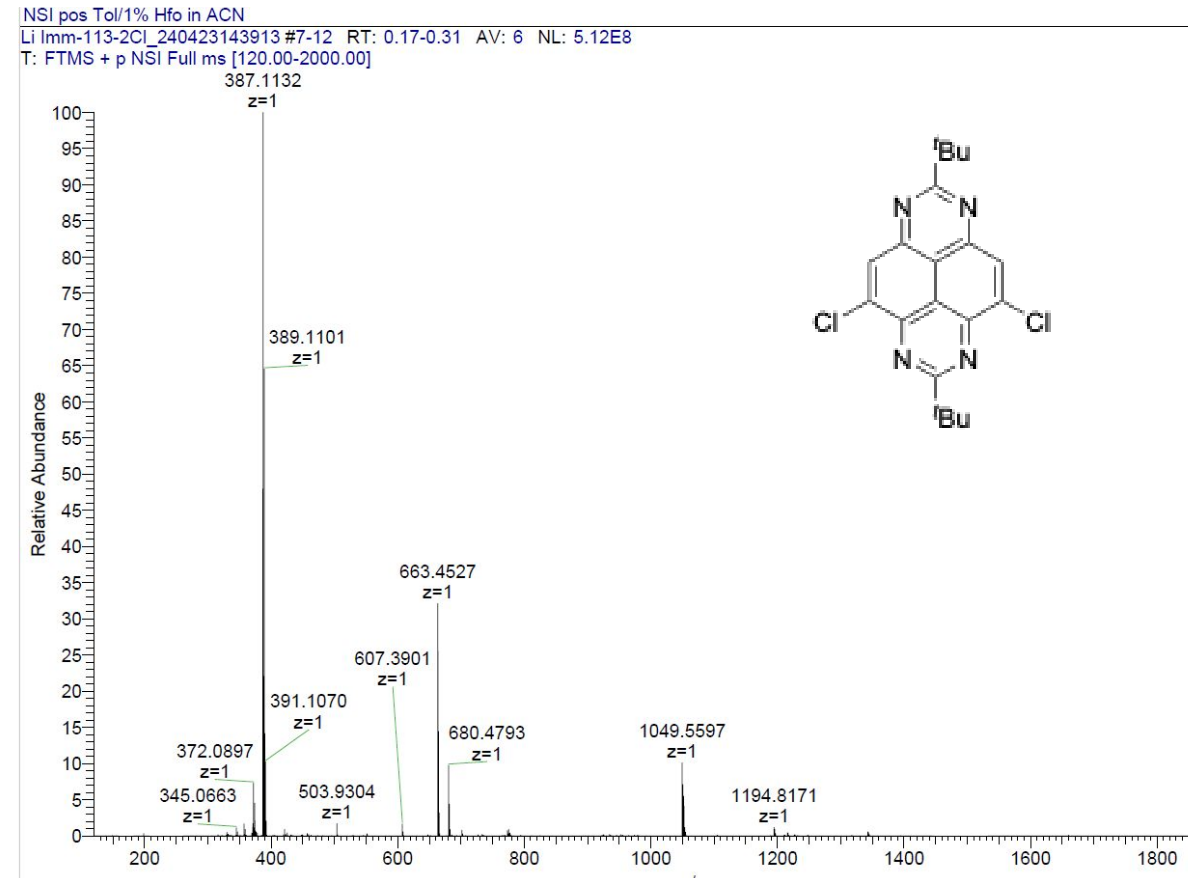

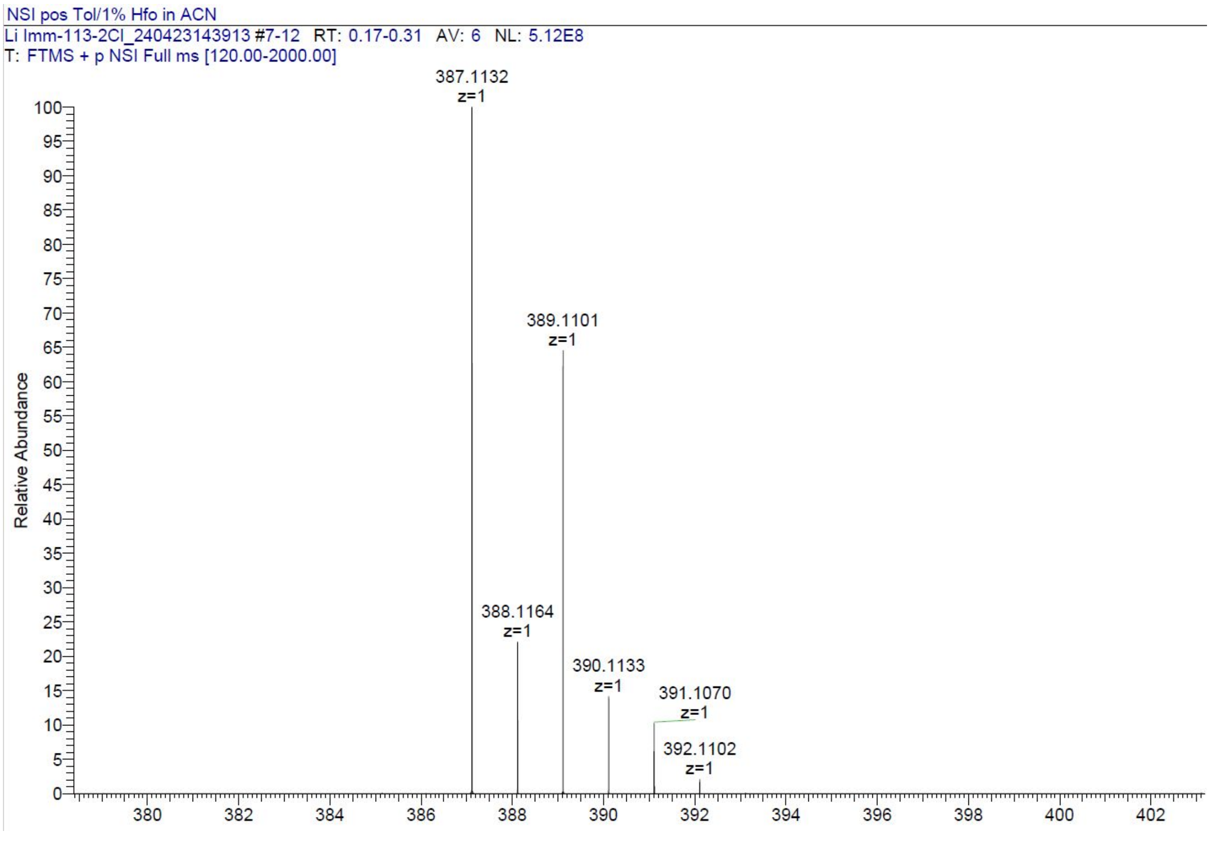

HR-MS spectrum of **4,10-Cl$_2$-^{t}Bu-TAP**.

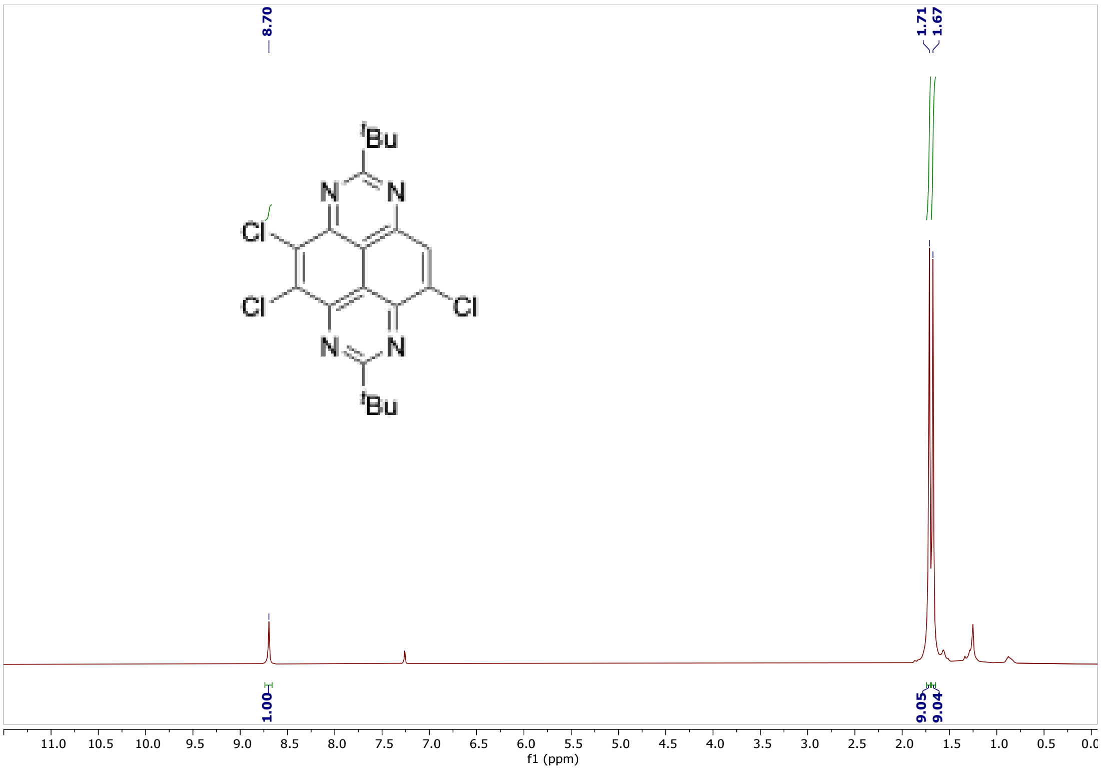

^{1}H NMR spectrum (400 MHz) of **Cl_3-^{t}Bu-TAP** in $CDCl_3$.

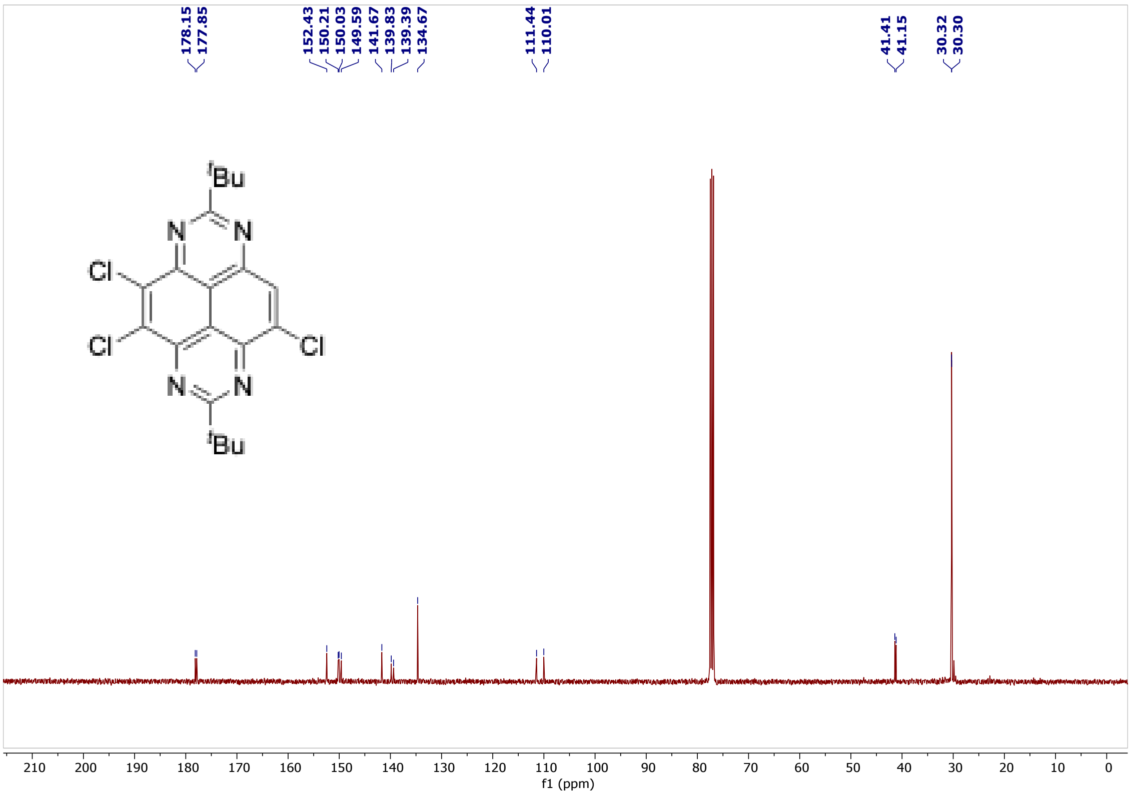

^{13}C NMR spectrum (101 MHz) of **Cl_3-^{t}Bu-TAP** in $CDCl_3$.

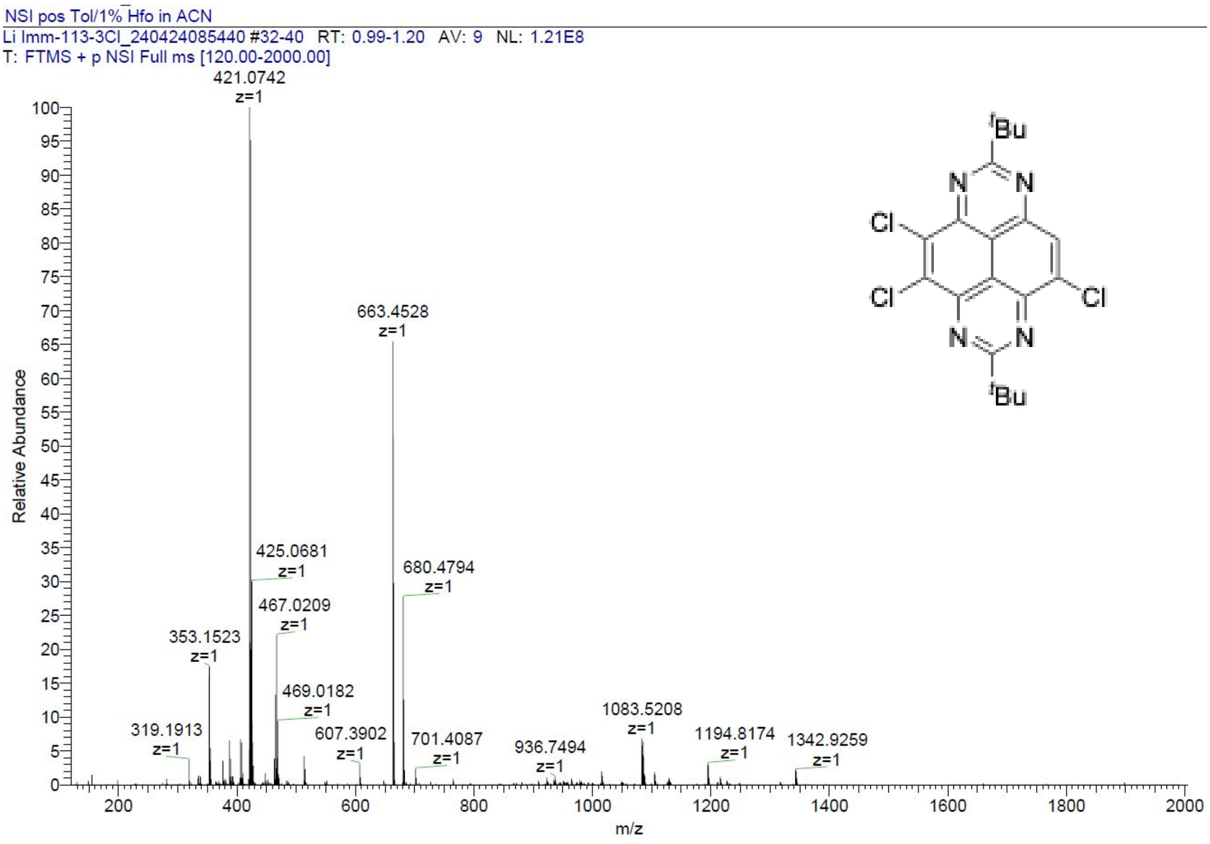

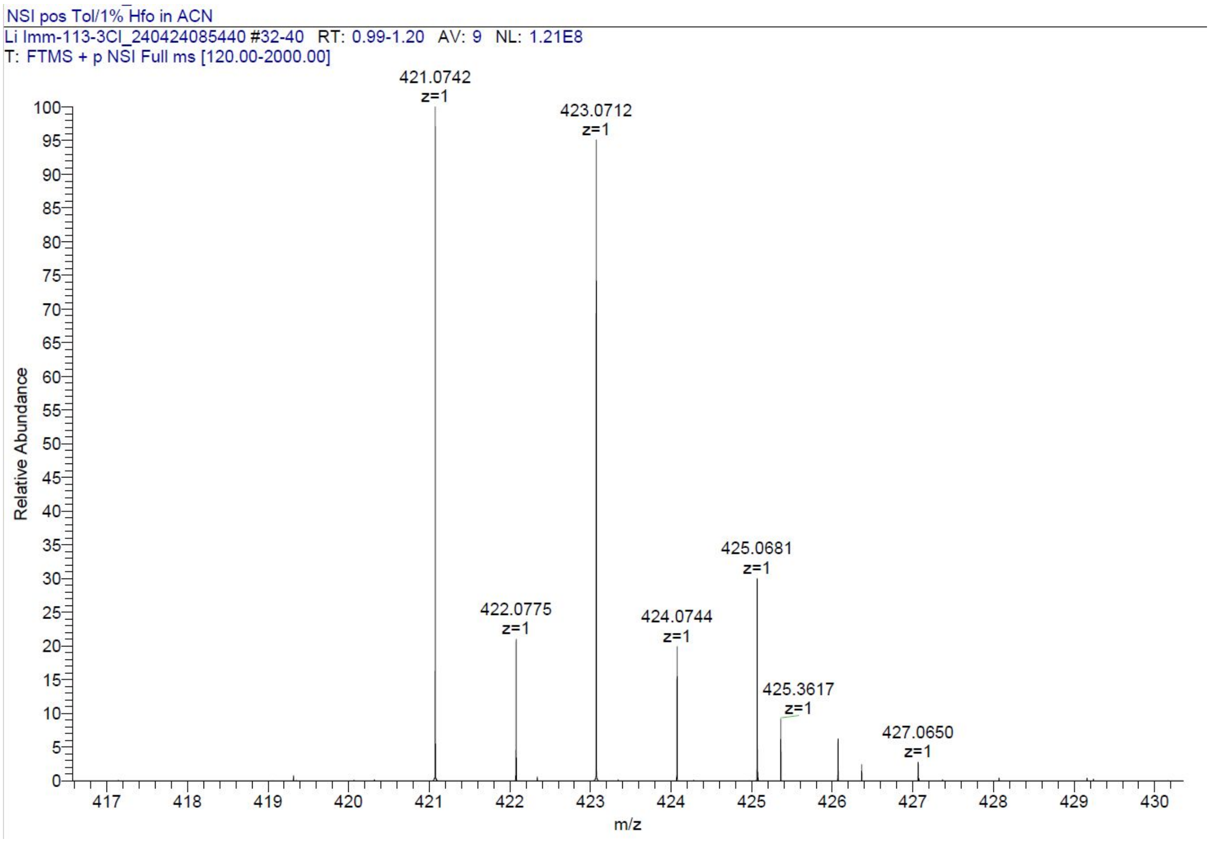

HR-MS spectrum of **Cl_3-^{t}Bu-TAP**.

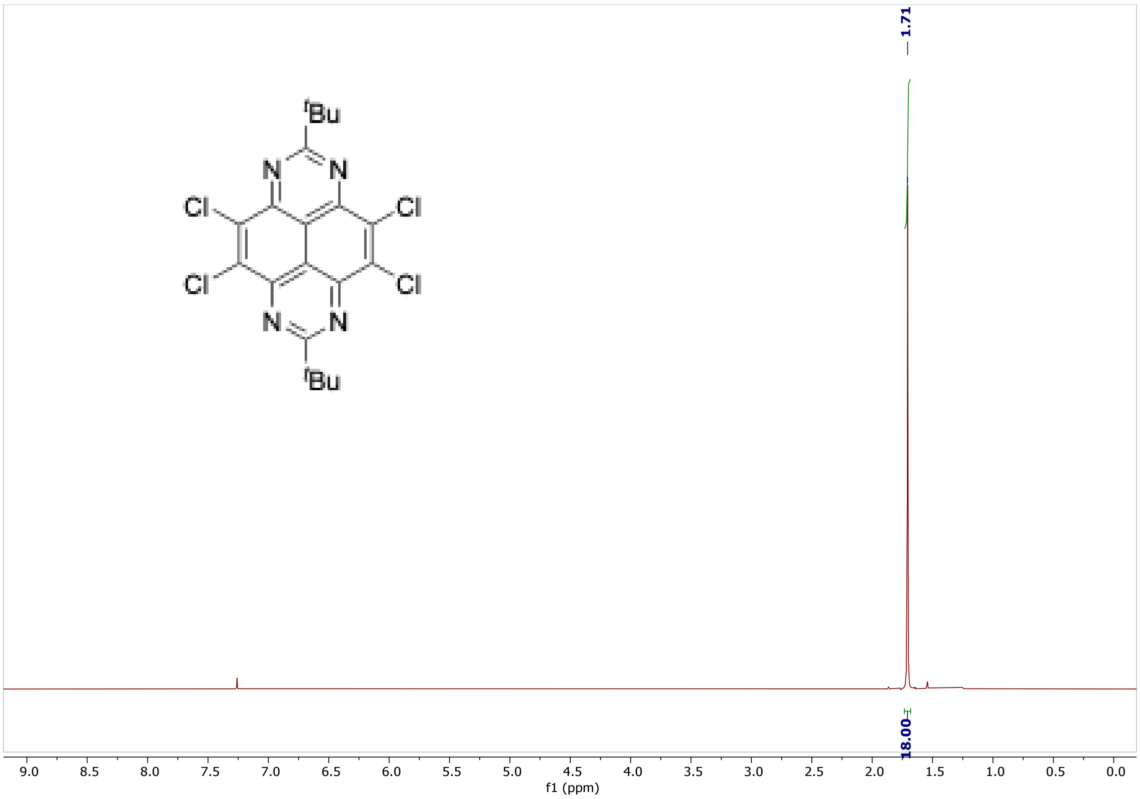

^{1}H NMR spectrum (400 MHz) of **Cl₄-*t*Bu-TAP** in $CDCl_3$.

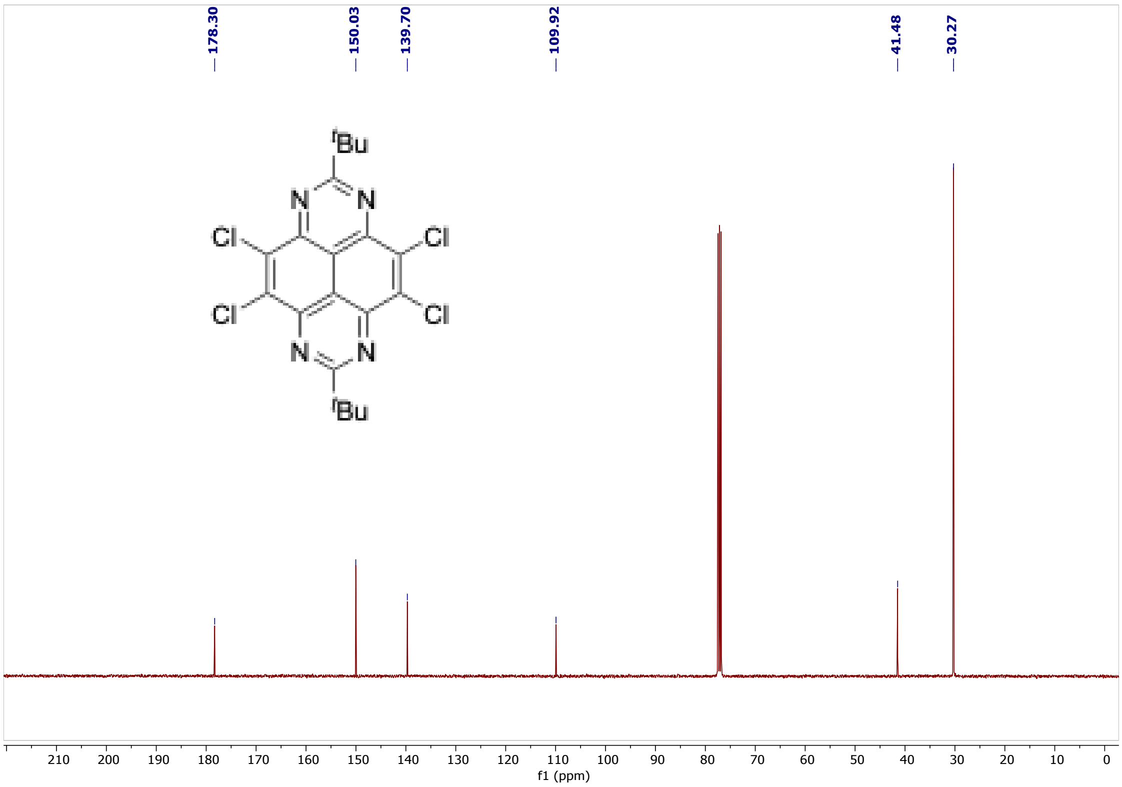

^{13}C NMR spectrum (101 MHz) of **Cl₄-*t*Bu-TAP** in $CDCl_3$.

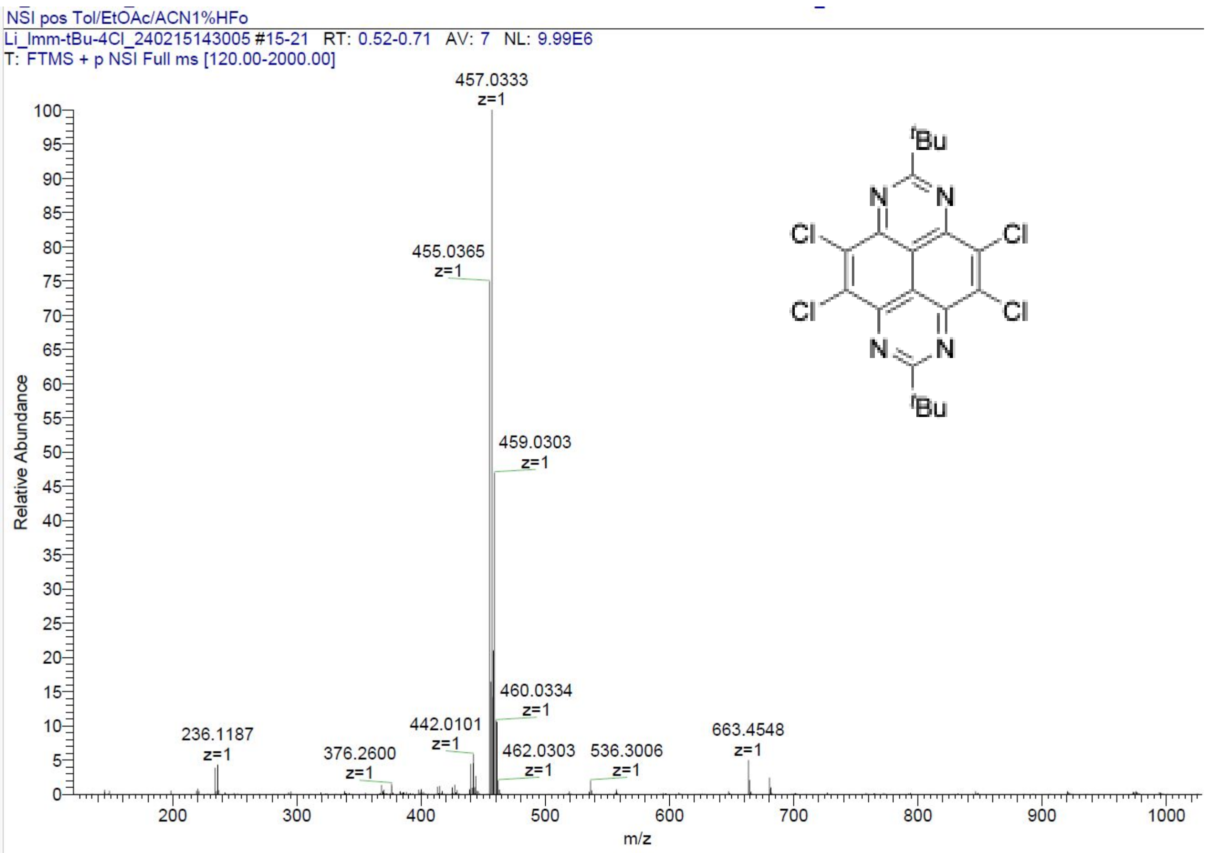

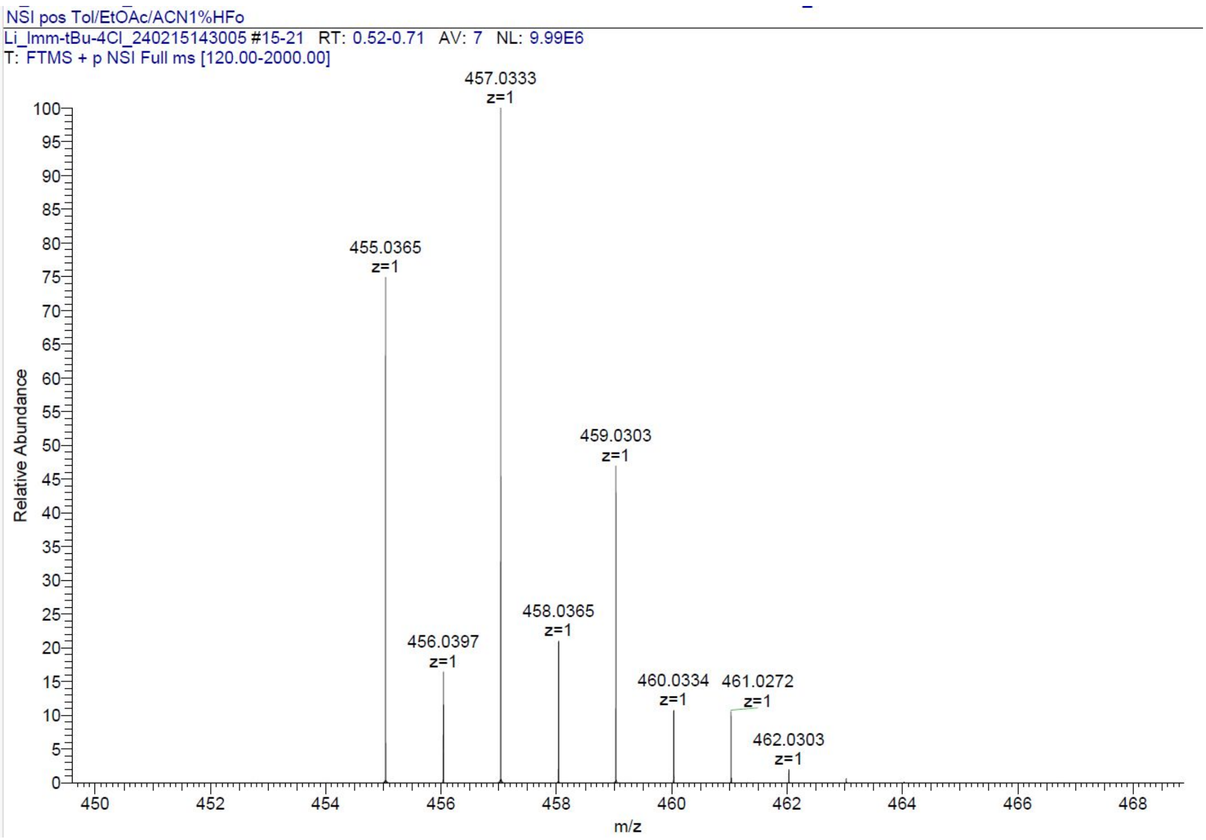

HR-MS spectrum of **Cl_4-^{t}Bu-TAP**.

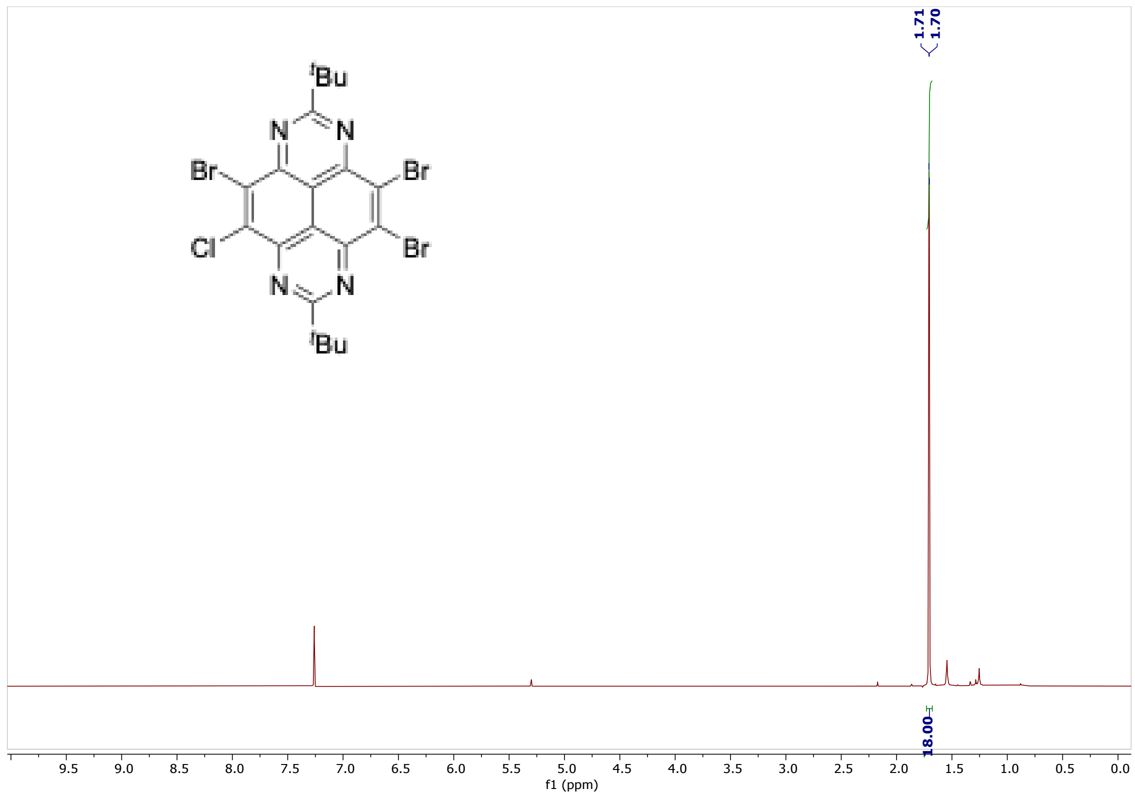

^{1}H NMR spectrum (400 MHz) of **Br$_3$Cl-^{t}Bu-TAP** in $CDCl_3$

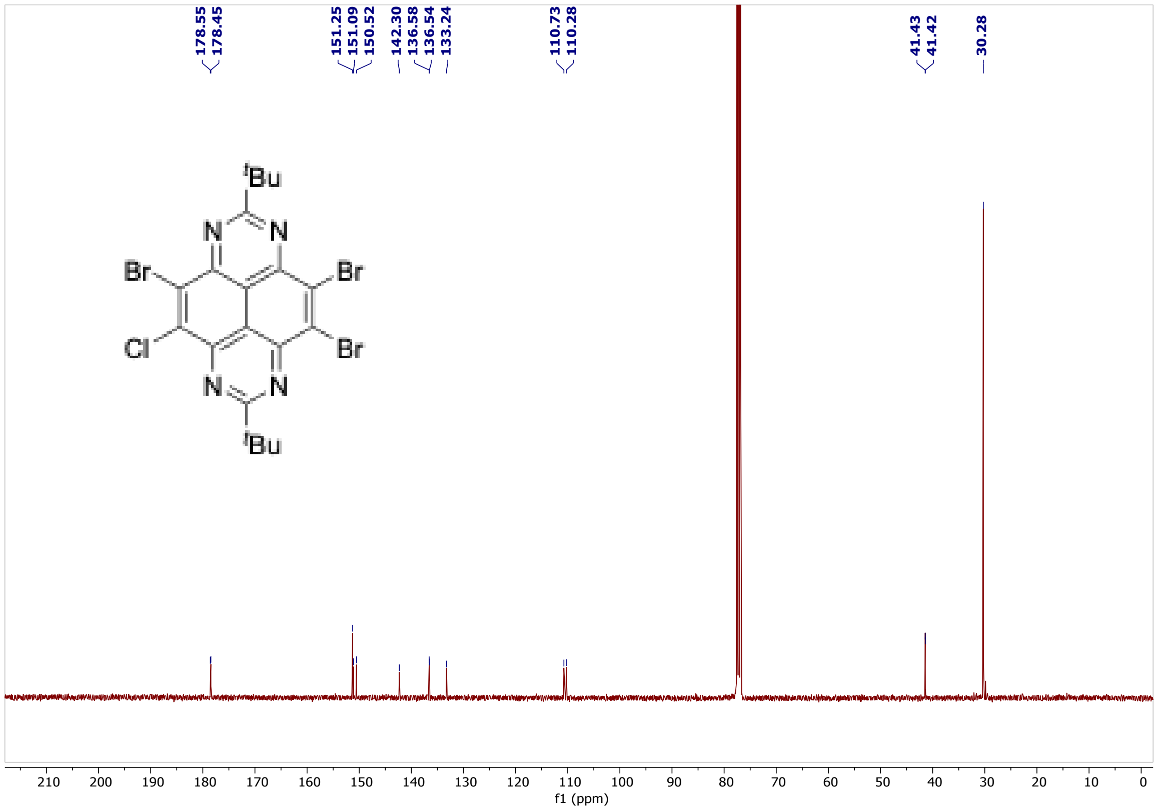

^{13}C NMR spectrum (101 MHz) of **Br$_3$Cl-^{t}Bu-TAP** in $CDCl_3$.

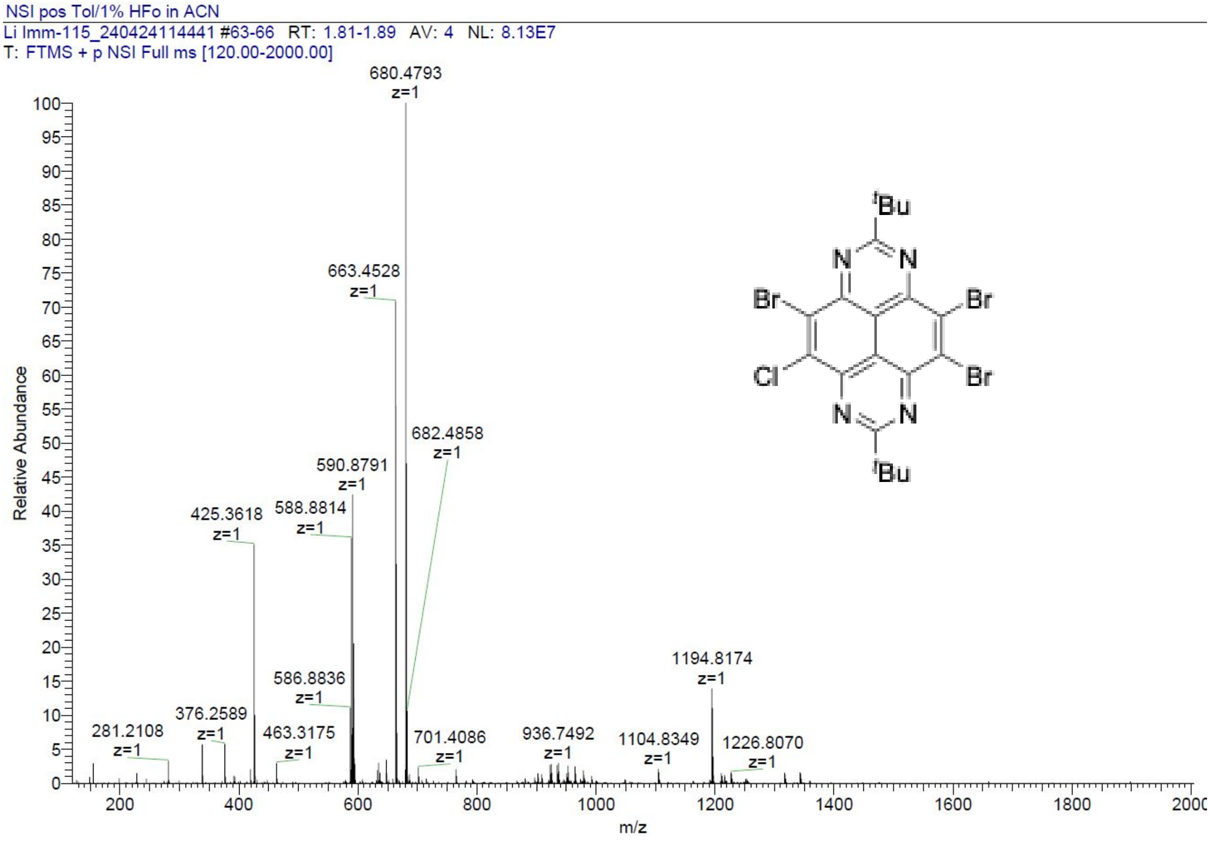

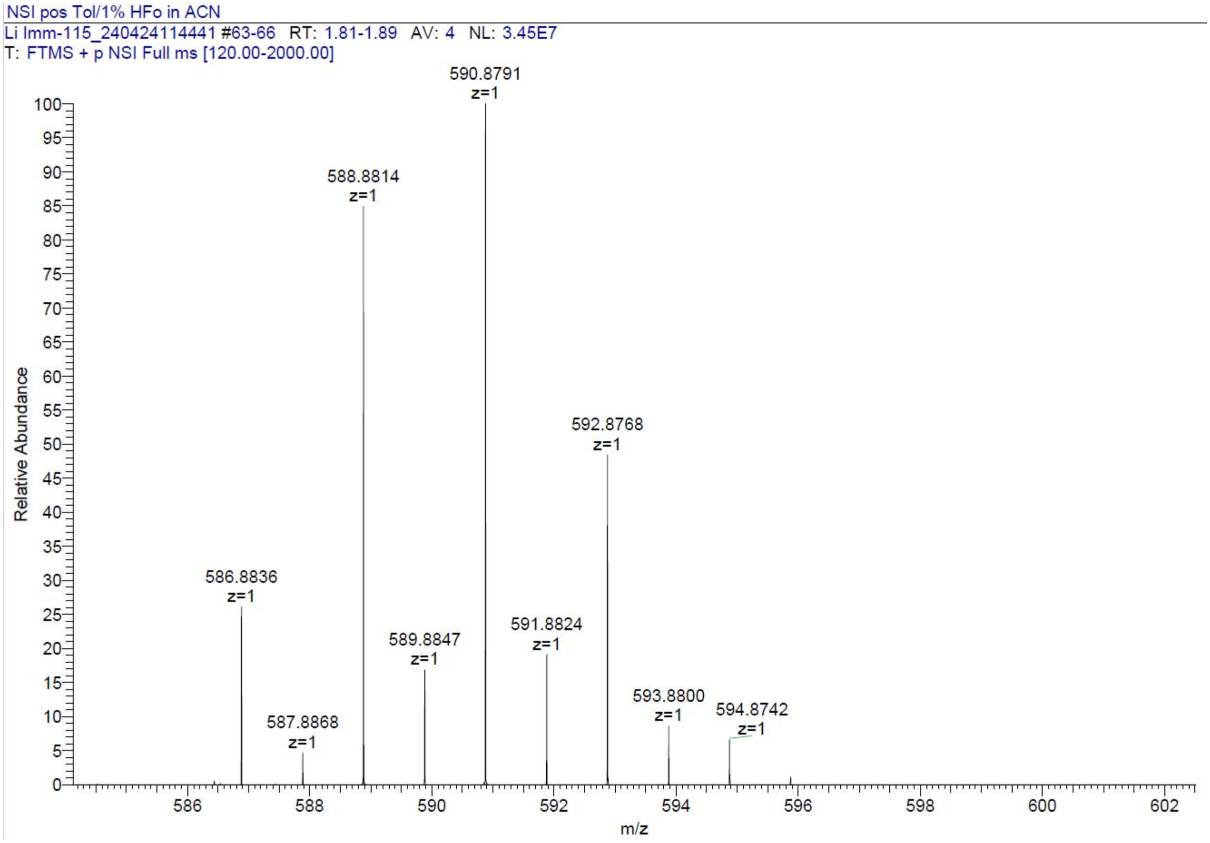

HR-MS spectrum of **Br_3Cl-^{t}Bu-TAP**.

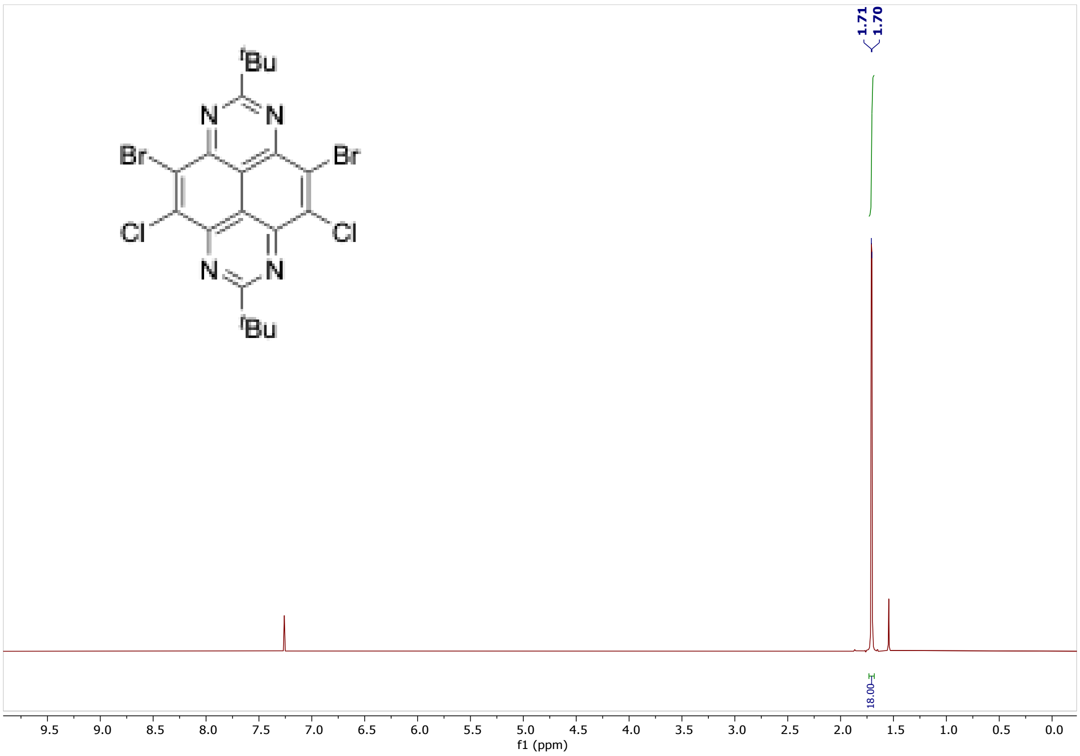

^{1}H NMR spectrum (400 MHz) of **Br_2Cl_2-*t*Bu-TAP** in $CDCl_3$.

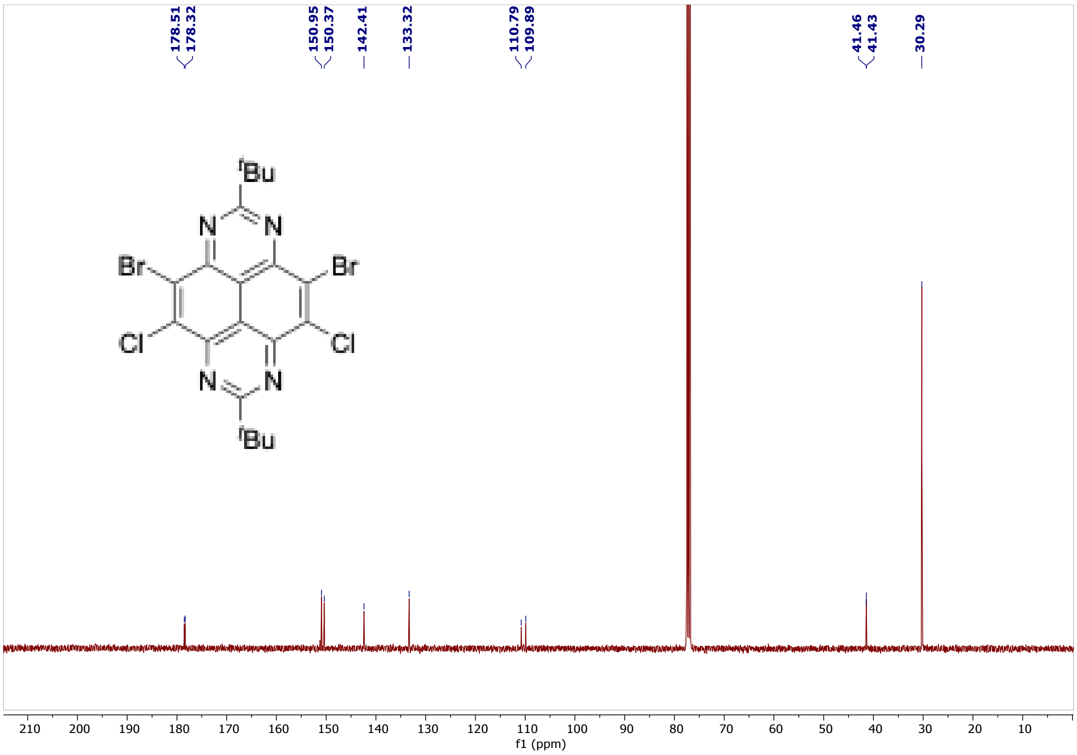

^{13}C NMR spectrum (101 MHz) of **Br_2Cl_2-*t*Bu-TAP** in $CDCl_3$.

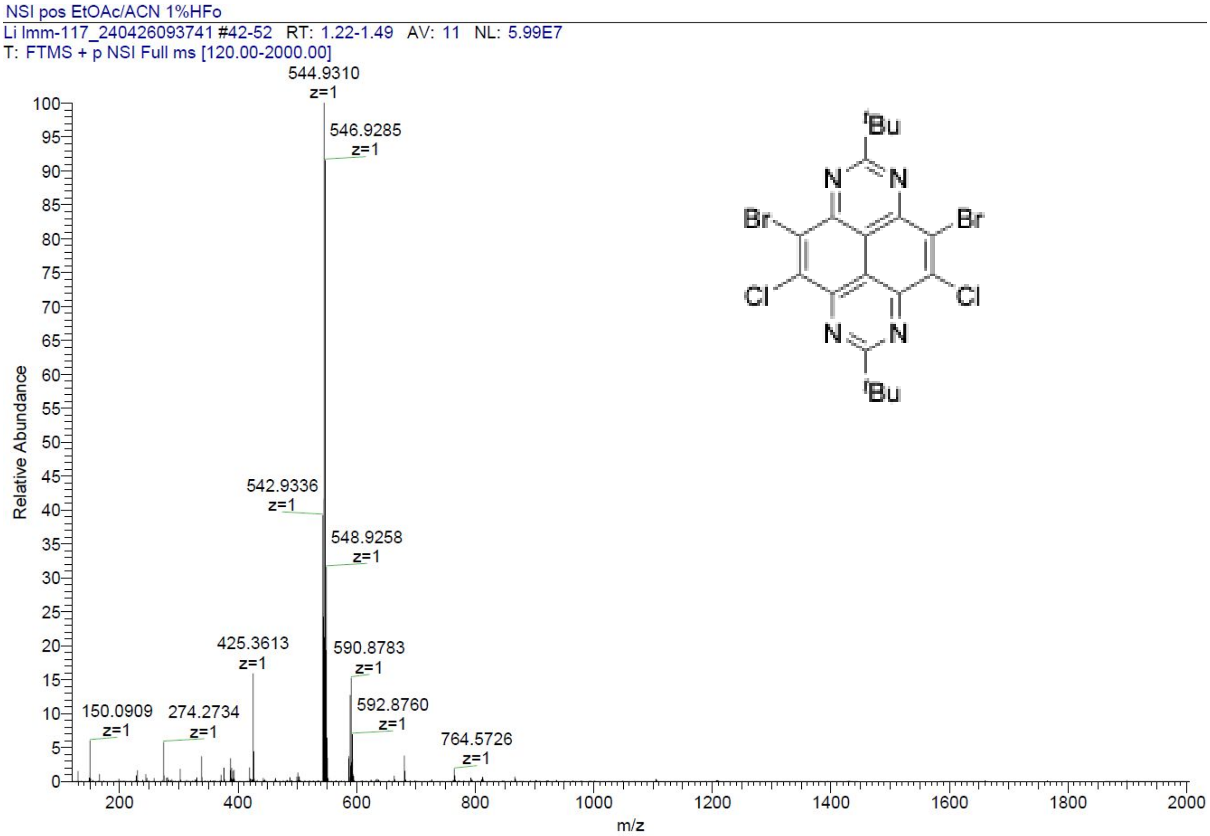

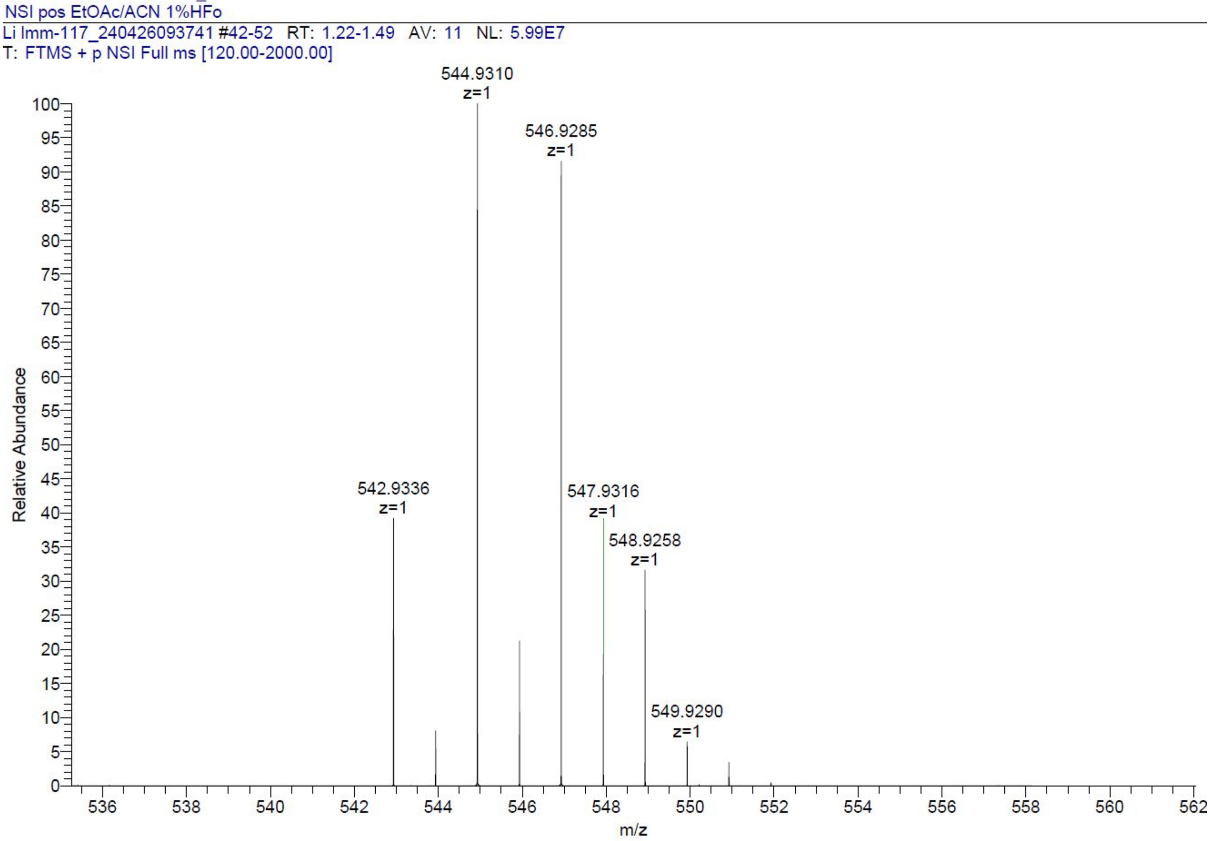

HR-MS spectrum of **Br_2Cl_2-^{t}Bu-TAP**.

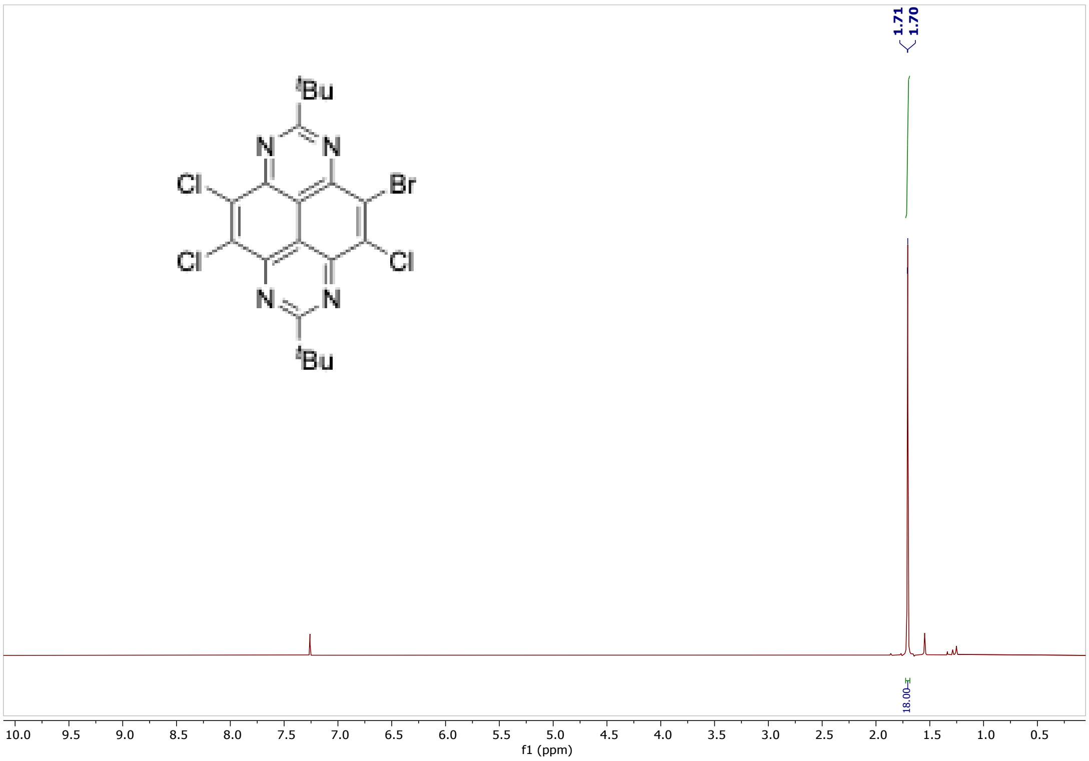

^{1}H NMR spectrum (400 MHz) of **BrCl$_3$-^{t}Bu-TAP** in CDCl$_3$.

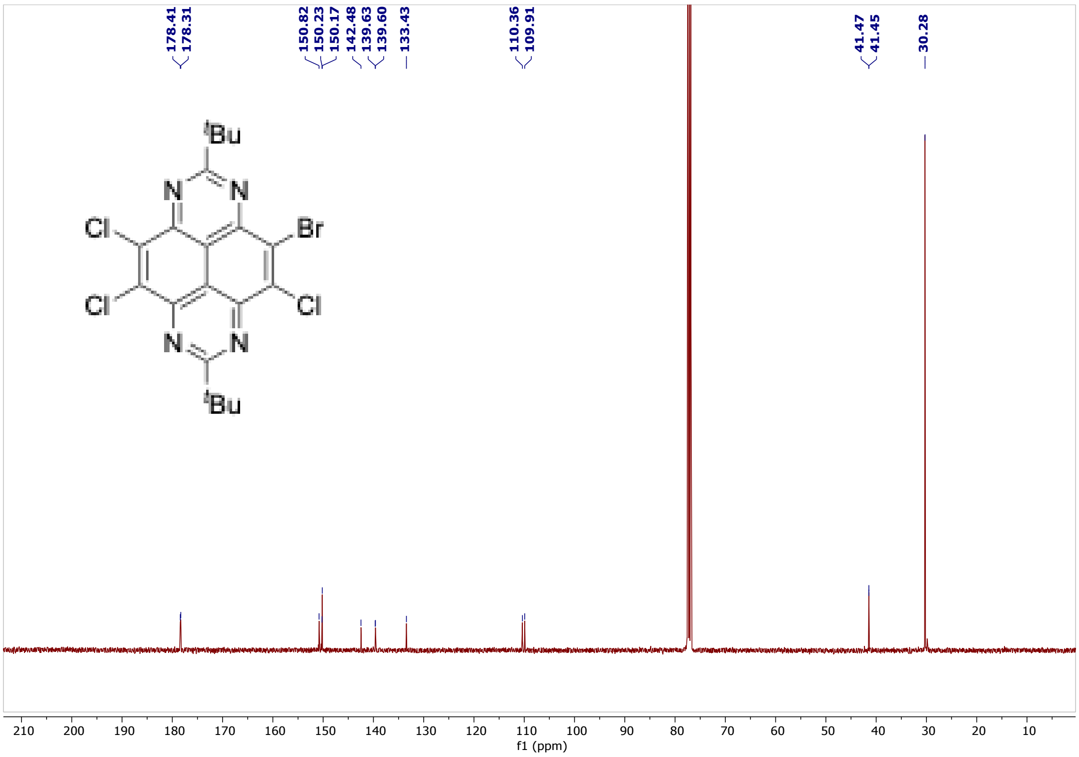

^{13}C NMR spectrum (101 MHz) of **BrCl$_3$-^{t}Bu-TAP** in CDCl$_3$.

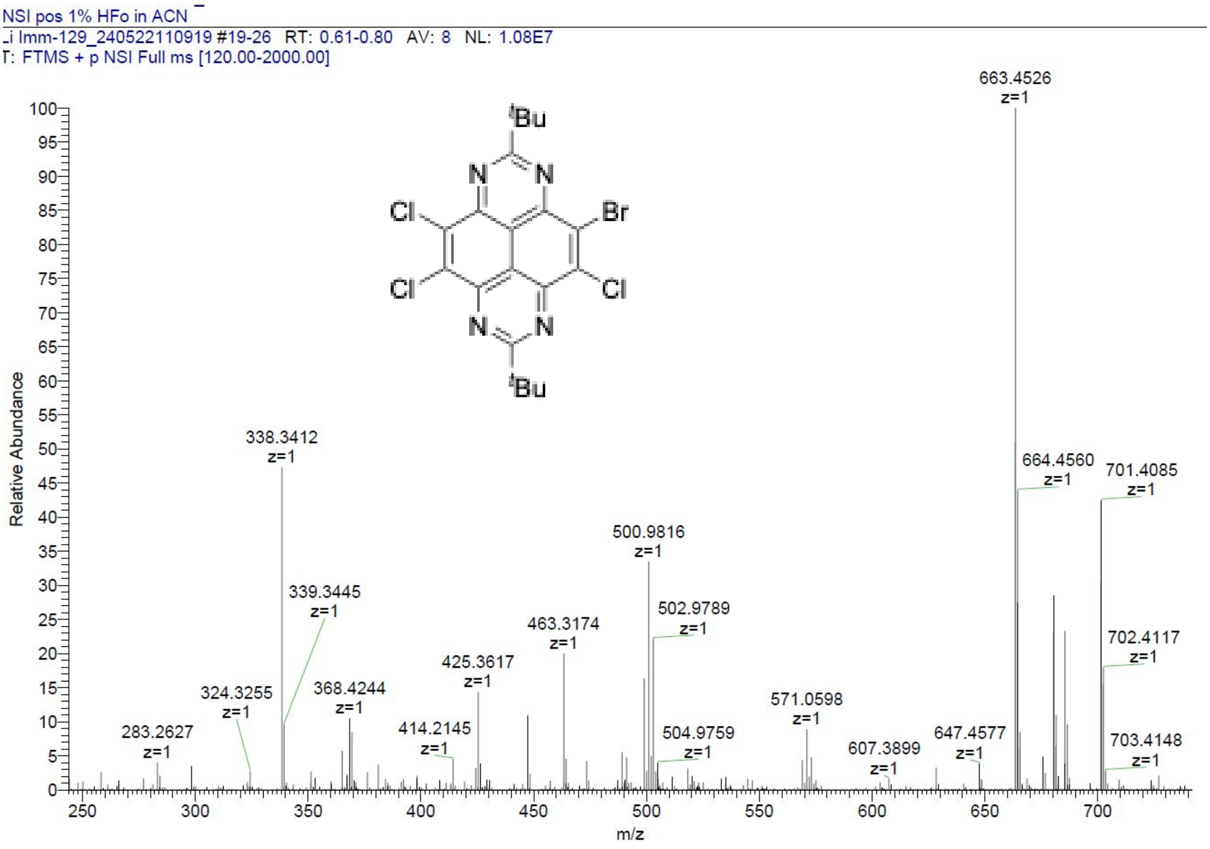

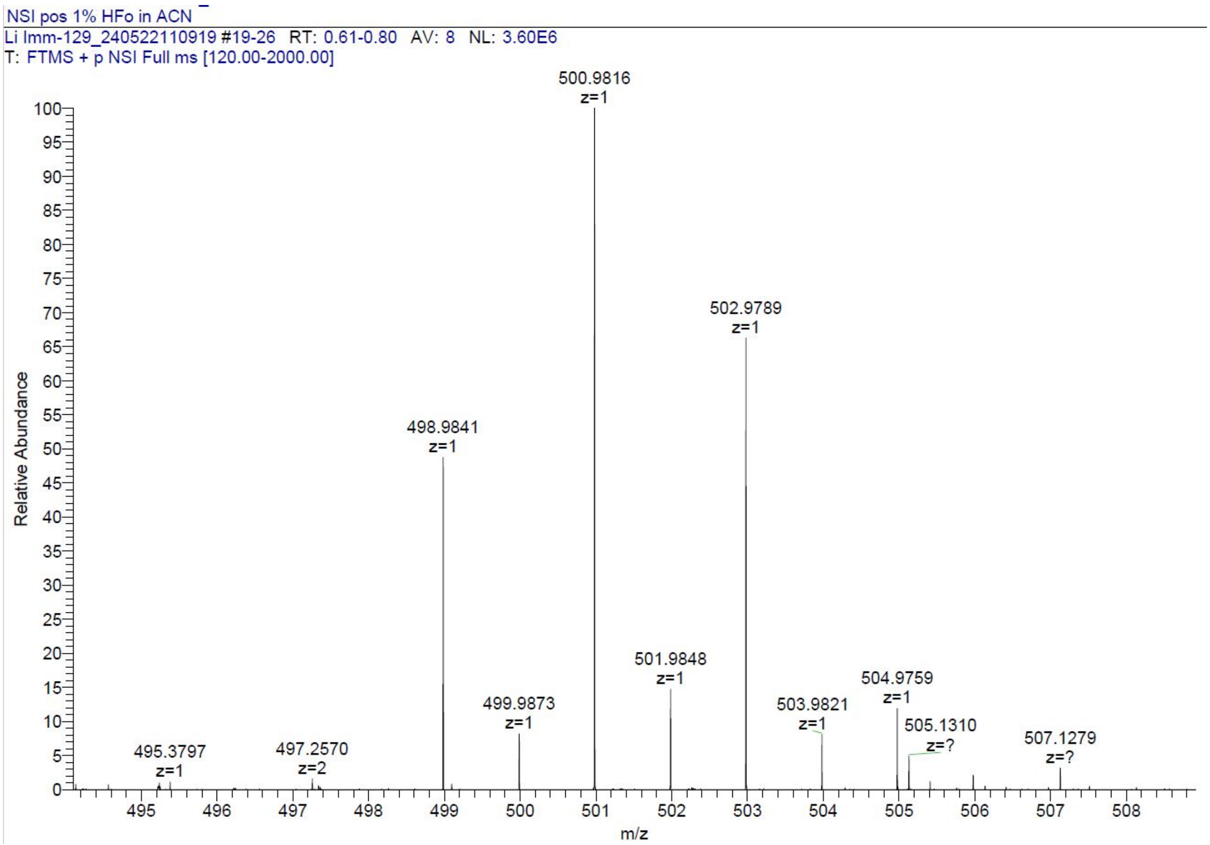

HR-MS spectrum of **BrCl$_3$-^{t}Bu-TAP**.

References

1. (a) P. Zhou, U. Aschauer, S. Decurtins, T. Feurer, R. Haner and S.-X. Liu, *Chem. Commun.* **2021**, *57*, 12972–12975; (b) Zhou, P.; Nazari Haghighi Pashaki, M.; Frey, H.-M.; Hauser, A.; Decurtins, S.; Cannizzo, A.; Feurer, T.; Haner, R.; Aschauer, U.; Liu, S.-X., *Chem. Sci.* **2023,** *14*, 12715-12722.
2. Oxford Diffraction (2018). *CrysAlisPro* (Version 1.171.40.37a). Oxford Diffraction Ltd., Yarnton, Oxfordshire, UK.
3. Sheldrick, G. M. (2015). *Acta Cryst*. **A71**, 3-8.
4. Sheldrick, G. M. (2015). *Acta Cryst*. **C71**, 3-8.
5. Dolomanov, O.V., Bourhis, L.J., Gildea, R.J, Howard, J.A.K. & Puschmann, H. (2009), J. Appl. Cryst. 42, 339-341.
